# Microphase Separation and Temperature-Dependent Structural Transitions of Mixed-Side-Chain Comb-like Copolymers

**DOI:** 10.3390/ma19183955

**Published:** 2026-09-17

**Authors:** Yuan-Hao Yao, Jun-Ting Xu, Ze-Kun Zhang

**Affiliations:** 1 State Key Laboratory of Biobased Transportation Fuel Technology, Department of Polymer Science and Engineering, Zhejiang University, Hangzhou 310058, China; xujt@zju.edu.cn; 2 Dalian Changfeng Industrial Corporation, Dalian 116038, China

**Keywords:** charged mixed-side-chain comb-like copolymer, microphase separation, lower disorder–order transition, Frank–Kasper A15, sub-5 nm structure

## Abstract

Introducing charged groups into comb-like copolymers significantly enhances the incompatibility between the main chain and side chains, enabling sub-5 nm microphase separation. In this work, a series of charged comb-like copolymers with imidazolium ionic junctions and mixed alkyl side chains (C18/C8, C20/C12, C22/C12) were synthesized. The regulation of microphase separation behavior via side-chain composition and length were investigated. The crystallization of long alkyl side chains could disrupt pre-formed microphase-separated structures, rendering a disordered state at low temperatures. The systems underwent a lower disorder–order transition (LDOT) that was closely correlated with the melting of side-chain crystallites, forming lamellar (LAM) or hexagonally packed cylindrical (HEX) nanostructures. By adjusting the side-chain length and feed ratio (*r*) of long/short chains, both the LDOT and closed-loop sequence of transitions (LDOT at low temperatures and upper order–disorder transition (UODT) at high temperatures) were achieved, with transition temperatures shifting accordingly. Notably, the SAXS data at a specific composition are consistent with the Frank–Kasper (F-K) A15 complex spherical phase. Moreover, the domain spacing showed an approximately linear dependence on the nominal long-chain feed ratio within the investigated range. This work demonstrates that statistical side-chain functionalization is an effective strategy for tuning microphase separation in charged comb-like copolymers with ultra-small self-assembled dimensions.

## 1. Introduction

Block copolymers (BCPs) can spontaneously self-assemble into periodically ordered nanostructures with tunable dimensions through microphase separation between chemically incompatible blocks, exhibiting broad application prospects in nanolithography, separation membranes, energy storage, and conversion [1,2,3,4,5,6]. Practical integration of these materials into commercial devices, however, requires overcoming additional challenges such as large-area film fabrication, domain orientation control, and processing compatibility. The present study focuses on the fundamental understanding of phase behavior as a necessary foundation for addressing these challenges.

The microphase separation behavior of AB diblock copolymers is primarily governed by the segregation strength *χN* and the block volume fraction *f* [7], where *χ* is the Flory–Huggins interaction parameter between the two blocks and *N* is the total degree of polymerization (*DP*) of BCPs. When *χN* is too small, only a disordered structure will be formed [8,9]. Moreover, the feature dimensions of the ordered structures of BCPs are proportional to *N* [10]. As the microelectronics industry increasingly demands sub-10 nm and even sub-5 nm feature sizes in nanopatterns, the development of “high-*χ*–low-*N*” BCPs has become a research hotspot [11]. However, conventional linear BCPs face a dilemma when pursuing ultra-small dimensions: reducing *N* can decrease the domain spacing, but high *χ* constituent blocks should be used to maintain ordered nanostructures, which usually involves complicated synthesis and polymerization of specially designed monomers. On the other hand, the *DP* of polymers synthesized in different batches cannot be exactly the same, as even the polymerization conditions are kept the same. Therefore, the domain spacing of the microphase-separated structures will vary with the synthesis batch more or less. In addition, the “high-*χ*–low-*N*” BCPs usually have a lower order–disorder transition temperature (*T*_ODT_) due to the low *DP*s, which leads to poor thermal stability of the microphase-separated structures and is disadvantageous to thermal processing.

Comb-like or bottlebrush polymers offer a new avenue to address this dilemma of BCPs due to their unique topological structures. In these polymers with high side-chain density, microphase separation is primarily driven by the incompatibility between the side chains and the main chain or between different side chains, and the domain spacing is primarily determined by the side-chain length rather than the *DP* of the mainchain, thereby decoupling dimension control of the nanostructures from molecular weight. Reported comb-like homopolymers include poly(*n*-alkyl methacrylates) (P*n*AMA) and *N*-alkylated polyethylenimine [12,13,14,15,16]. For example, Hempel et al. found that for the chemical incompatibility between the polar polymethacrylate main chains and the nonpolar *n*-alkyl side chains in P*n*AMA homologues could induce nanophase separation, and the domain spacing increased with increasing the side-chain length [16]. In addition, mixed-side-chain copolymerization is usually utilized to regulate the structure of this type of polymer. For example, Ikami et al. reported that amphiphilic random copolymers bearing octadecyl and hydrophilic hydroxyethyl side groups could form multilayered lamellar structures in bulk and thin films, and the domain spacing (3.1–4.2 nm) was precisely tunable at the 0.1 nm level by adjusting side-chain length or octadecyl content [17].

It should be noted that the microphase separation strength between the side chains and the main chain in non-charged comb-like polymers is relatively weak due to the limited length of the side chains. As a result, other factors, such as crystallization of the side chain, are usually introduced to enhance the microphase separation strength [12,16]. However, once the temperature rises above the melting temperature of the side-chain, the ordered nanostructures will rapidly disintegrate, severely limiting the application of these materials over a broad temperature window. Sujita et al. also prepared alternating copolymers bearing both imidazolium cations and hydrophobic alkyl side chains, and the cationic groups could absorb moisture from the environment, thereby driving side-chain self-assembly [18]. It was demonstrated that both morphology and domain spacing (2.8–5.1 nm) were governed by the hydrophobic side-chain structure and the amount of absorbed water, achieving sub-0.01 nm tunability.

In addition, it is found that the introduction of charged groups at the junctions between two blocks in BCPs or between the side chain and main chain in comb-like polymers can greatly enhance the microphase separation strength [19,20,21,22]. For example, a 1,2,3-triazolium ionic junction was introduced into the poly(dimethylsiloxane)-*b*-poly(methyl methacrylate) (PDMS-*b*-PMMA) and PDMS-*b*-poly(ethylene oxide) (PEO) to enhance the segregation strength of BCPs [19]. Our previous work demonstrated that the comb-like polymers, poly(1-((2-acryloyloxy)ethyl)-3-alkylimidazolium bromide) (P(AOEA*_m_*I-Br)) with imidazolium ionic junctions, could form ordered sub-5 nm structures with excellent thermal stability even when the side chains were in molten state, making them good candidates for advanced lithography. However, several aspects of this system remain to be clarified. The accessible phase morphologies are currently limited to simple LAM and HEX structures, and it is unclear whether more complex phase structures can be achieved by further tuning the molecular parameters. Additionally, the role of side-chain crystallization in the microphase-separation process—particularly whether it acts as a cooperative or competing factor in the presence of strong ionic interactions—has not been systematically elucidated.

To address these issues, in this work, we designed and synthesized a series of charged mixed-side-chain comb-like copolymers featuring imidazolium ionic junctions and side chains composed of alkyl chains of varying lengths (C18/C8, C20/C12, C22/C12) to modulate the crystallizability of the side chains. The long-chain length was progressively extended from C18 to C22 to systematically enhance the crystallizability and segregation strength, while the short chains (C8 and C12) were kept non-crystallizable, allowing us to evaluate the role of side-chain crystallization in the phase behavior. Meanwhile, it is expected that mixed-side-chain functionalization also enables effective tuning of the volume fractions of the side chains and the main chain, providing a convenient method for precisely controlling the domain spacing and phase structure. Therefore, the microphase separation behavior of these charged mixed-side-chain comb-like copolymers with different side chain pairs and compositions was systematically investigated.

## 2. Materials and Methods

### 2.1. Materials

Azobisisobutyronitrile (AIBN, 98%, Sigma-Aldrich, St. Louis, MO, USA) was recrystallized twice from methanol and stored at −20 °C. 2-Bromoethanol (97%, J&K Scientific, Beijing, China), 1-bromododecane (98%, Aladdin, Shanghai, China), 1-bromooctadecane (98%, J&K Scientific), 1-bromoeicosane (98%, J&K Scientific), 1-bromodocosane (98%, Meryer, Shanghai, China), 1-octylimidazole (99%, Macklin, Shanghai, China), imidazole (99%, Aladdin), acrylic acid (99%, Sinopharm Chemical Reagent, Shanghai, China), 4-dimethylaminopyridine (DMAP, 99%, Macklin), and 1-ethyl-3-(3-dimethylaminopropyl)carbodiimide hydrochloride (EDCI, 99%, Dibai, Shanghai, China) were used as received. *N*,*N*-Dimethylformamide (DMF, 99%, Sinopharm Chemical Reagent), tetrahydrofuran (THF, 99%, Sinopharm Chemical Reagent), dichloromethane (99%, Sinopharm Chemical Reagent), chloroform (99%, Sinopharm Chemical Reagent), *n*-hexane (99%, Tianjin Fangping Chemical Reagent, Tianjin, China), and 2-butanone (99%, Yonghua Chemical Technology, Suzhou, China) were of analytical grade and used as received. Deuterated dimethyl sulfoxide (DMSO-d_6_, J&K Scientific) was used as the solvent for NMR measurements.

### 2.2. Synthesis of C18_x_C8_y_B_z_

**Scheme 1:**
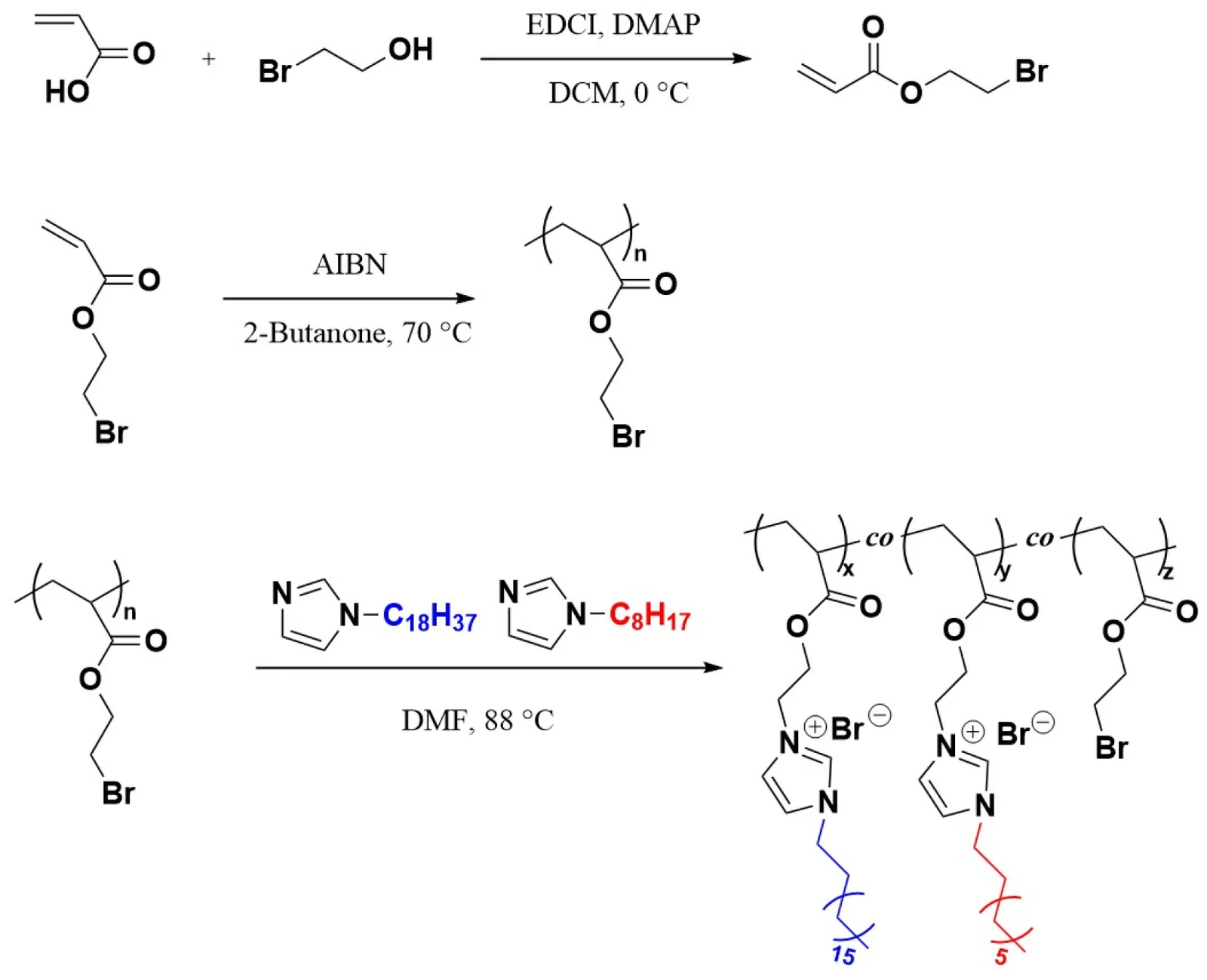
Synthetic routes of C18*_x_*C8*_y_*B*_z_* samples.

Scheme 1 shows the synthetic route for the mixed-side-chain comb-like copolymers. The synthesis involves two stages: first, poly(2-bromoethyl acrylate) (PBrEA) homopolymers were prepared via solution free-radical polymerization; second, quaternization modification of PBrEA was performed using a mixture of 1-octylimidazole and 1-octadecylimidazole at various feed ratios (*r*), yielding the target copolymers. The products are denoted as C18*_x_*C8*_y_*B*_z_*, where the subscripts *x*, *y* represent the molar fractions of repeat units bearing octadecyl side-chain or octyl side-chain, and the subscript *z* represents the molar non-quaternized 2-bromoethyl acrylate (BrEA) repeating units, respectively. Detailed synthetic procedures and characterizations of the products at each step (including ^1^H-NMR and ^13^C-NMR spectra and GPC traces) are provided in the Supplementary Materials (Figures S1–S19, Table S3).

### 2.3. Synthesis of C20_r_C12_1-r_ and C22_r_C12_1-r_

Similarly, by replacing the long-chain alkylimidazole units with 1-eicosylimidazole or 1-docosylimidazole and the short-chain alkylimidazole units with 1-dodecylimidazole and varying the feed ratio *r* of long- to short-chain alkylimidazole units, a series of C20*_r_*C12_1-_*_r_* and C22*_r_*C12_1-_*_r_* charged mixed-side-chain copolymer samples were obtained via the quaternization of the PBrEA precursor under the same reaction and work-up conditions as for the C18*_x_*C8*_y_*B*_z_* series. (Owing to the limited solubility of samples with high long-chain alkyl content in conventional deuterated solvents (Figures S20 and S21), the samples are denoted by their nominal feed ratios. The reader should note that *r* serves as a nominal parameter, which may slightly deviate from the absolute copolymer composition due to potential differences in quaternization efficiency.) Detailed synthetic procedures are provided in the Supplementary Materials.

### 2.4. Characterizations

The chemical structures and compositions of the polymers were characterized by nuclear magnetic resonance spectroscopy (NMR). The molecular weights and molecular weight distributions of the PBrEA precursors were determined by gel permeation chromatography (GPC). The thermal transitions and crystallization behaviors of the samples were investigated by differential scanning calorimetry (DSC) and polarized optical microscopy (POM). The microphase-separated structures and their temperature-dependent evolutions were characterized by synchrotron small-angle X-ray scattering (SAXS). The water content of the polymers was determined by thermogravimetric analysis (TGA) based on the weight loss at elevated temperatures. Detailed experimental procedures and parameters are described below.

^1^H-NMR spectra were recorded on a Bruker DMX-400 spectrometer (Bruker, Billerica, MA, USA) at room temperature. DMSO-d_6_ was used as the solvent at a concentration of approximately 8.3 mg/mL, with tetramethylsilane (TMS) as the internal standard. The quaternization degree and molar fractions of each structural unit were calculated from the integral ratios of characteristic peaks.

^13^C-NMR spectra were recorded on a JNM-ECZL400S spectrometer (JEOL, Tokyo, Japan) at room temperature. CDCl_3_ was used as the solvent at a concentration of approximately 60 mg/mL, with TMS as the internal standard. An inverse-gated decoupling sequence was employed to suppress the Nuclear Overhauser Effect, and the structure of the alkylimidazole intermediate was determined from the integral ratios of the characteristic carbon signals.

The molecular weights and distributions of the non-quaternized PBrEA precursors were determined using a Waters GPC system (Waters, Milford, MA, USA) equipped with a 1515 HPLC pump (Waters, Milford, MA, USA) and a 2414 refractive index detector (Waters, Milford, MA, USA). THF was used as the mobile phase at a flow rate of 1.0 mL/min, and the measurements were performed at 30 °C with a sample concentration of 0.5 wt%. Molecular weights were calibrated against monodisperse polystyrene standards (Figure S2, Table S1).

Thermal transitions of the polymers were measured using a TA Q200 differential scanning calorimeter (TA Instruments, New Castle, DE, USA). Approximately 5 mg of sample was sealed in an aluminum pan and measured under a nitrogen atmosphere. The thermal protocol was as follows: first cooled from room temperature to −20 °C at 10 °C/min, then held isothermally for 3 min, then heated to 100 °C at 10 °C/min. The heating curve was recorded for analysis.

TGA was performed on a TA Instruments SDT 650 (TA Instruments, New Castle, DE, USA) from room temperature to 140 °C at a heating rate of 10 °C/min under N_2_ atmosphere.

POM observations were performed on an Olympus BX51 microscope (Olympus, Tokyo, Japan) equipped with an INSTEC HCS402XY hot stage (Instec, Boulder, CO, USA). Samples were placed between a cover glass and a glass slide and heated at a rate of 10 °C/min. Polarized images were recorded at various temperatures.

Temperature-dependent SAXS experiments were conducted at beamline BL16B1 of the Shanghai Synchrotron Radiation Facility (SSRF, Shanghai, China). The X-ray wavelength *λ* was 1.24 Å, and the sample-to-detector distance was 2130 mm. Two-dimensional scattering patterns were recorded using a Pilatus 2M detector (Dectris, Baden-Daettwil, Switzerland), and SGTools software (Version 2.02) was used to convert the 2D patterns to 1D scattering profiles. Prior to measurement, samples were dissolved in anhydrous ethanol and annealed at 70 °C for 48 h with slow solvent evaporation, followed by vacuum annealing at 70 °C for 24 h. For temperature-dependent measurements, samples were placed on a Linkam hot stage (Linkam Scientific Instruments, Tadworth, UK) and heated to the pre-set temperature at 10 °C/min under a nitrogen atmosphere. After isothermal equilibration for 3 min, data were collected with an average exposure time of 20 s. It should be noted that while this heating protocol effectively captures the continuous structural evolution, the 3 min equilibration time at each temperature step may be insufficient for complex macromolecular/ionic architectures to reach absolute thermodynamic equilibrium. Therefore, the scattering data in this work represent temperature-dependent structural transitions during the heating process. The scattering vector *q* was calibrated using a silver behenate standard. Ordered structures were assigned based on the scattering vector ratios *q*/q* (positions of higher-order peaks relative to the primary peak): LAM phases correspond to 1:2:3…; hexagonally packed cylindrical (HEX) phases correspond to 1:√3:2:√7…, and the Frank–Kasper A15 phase was indexed according to the *Pm*$\bar{3}$*n* space group of the cubic crystal system [23]. The variations of the full width at half-maximum (FWHM) and the reciprocal intensity (1/Intensity) of the primary scattering peak with temperature were used to determine the disorder–order transition temperature (*T*_DOT_) and order–disorder transition temperature (*T*_ODT_) [24]. Peak fitting was performed using the built-in Lorentz function in Origin software (Origin 2021). A representative set of fitting parameters, including FWHM and peak height intensity, is provided in Table S4 in the Supplementary Materials.

## 3. Results and Discussion

### 3.1. Overall Temperature-Dependent Structural Transitions of Charged Mixed-Side-Chain Comb-like Copolymers

Before discussing the SAXS results in detail, it is useful to clarify the terminology used throughout this section. In this work, a sample is referred to as exhibiting “long-range order” when its SAXS profile shows a primary scattering peak accompanied by well-defined higher-order reflections that can be unambiguously indexed to a specific space group (e.g., LAM, HEX, or A15). Conversely, when only a broad primary peak is observed without discernible higher-order reflections, the sample is denoted as “DIS” (disordered). It should be emphasized, however, that the presence of a broad SAXS maximum still indicates a characteristic correlation length or local segregation and does not imply a completely homogeneous state. Thus, “DIS” in this context signifies a lack of long-range periodic order rather than the absence of nanoscale compositional fluctuations or local microphase separation.

**Figure 1:**
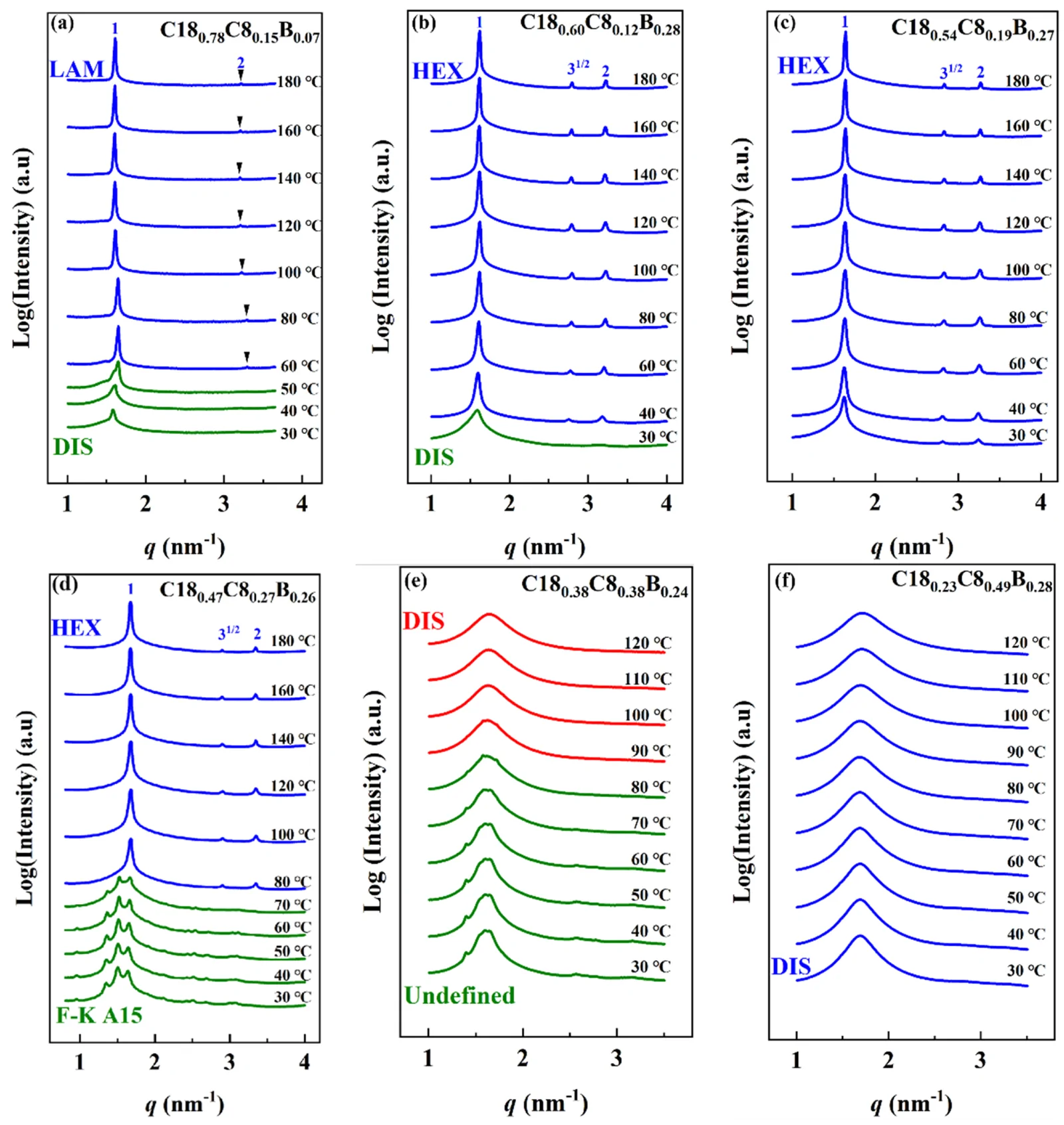
Temperature-variable SAXS profiles of (**a**) C18_0.78_C8_0.15_B_0.07_, (**b**) C18_0.60_C8_0.12_B_0.28_, (**c**) C18_0.54_C8_0.19_B_0.27_, (**d**) C18_0.47_C8_0.27_B_0.26_, (**e**) C18_0.38_C8_0.38_B_0.24_, (**f**) C18_0.23_C8_0.49_B_0.28_.

The phase behavior of the C18*_x_*C8*_y_*B*_z_* mixed-side-chain comb-like copolymers was first investigated, and their temperature-variable SAXS profiles are collected in Figure 1.

The sample C18_0.78_C8_0.15_B_0.07_ with the highest C18 content exhibits only a broad primary peak with no discernible higher-order peaks in the temperature range of 30–50 °C (Figure 1a), indicating a disordered state. However, upon heating to 60 °C, the primary scattering peak becomes much sharper suddenly and a second-order peak appears at 2q*, implying the formation of a LAM structure (Figure S22). Such a LAM structure remains stable even at 180 °C. Above results demonstrate that C18_0.78_C8_0.15_B_0.07_ is disordered at low temperatures, but it can transform into ordered stated upon heating. Therefore, C18_0.78_C8_0.15_B_0.07_ exhibits a lower disorder–order transition (LDOT) upon heating in the temperature range studied. Similarly, the LDOT is also observed for the sample C18_0.60_C8_0.12_B_0.28_ with slightly lower C18 content, being disordered at 30 °C but ordered at high temperatures (Figure 1b). As compared with C18_0.78_C8_0.15_B_0.07_, the LDOT of C18_0.60_C8_0.12_B_0.28_ occurs at a lower temperature (~30–40 °C), and the ordered structure in the high temperature range is HEX, which is confirmed by the higher-order scattering peaks at √3q* and 2q* (Figure S23). The origin of the LDOT will be discussed in the next subsection. When the C18 content is further decreased, C18_0.54_C8_0.19_B_0.27_ forms a HEX structure in the whole temperature range of 30–180 °C (Figure 1c and Figure S24), and no phase transition is observed. The sample C18_0.47_C8_0.27_B_0.26_ is ordered in the temperature range of 30–180 °C, but the SAXS pattern in 30–70 °C is consistent with a Frank–Kasper (F-K) A15 phase, which turns into a HEX structure when heated to higher temperatures. This indicates an order–order transition (OOT) occurs. The assignment of F-K A15 phase will be presented later. In contrast, a broad and strong scattering peak overlapped with a weak but sharp shoulder peak is observed for the SAXS profiles of C18_0.38_C8_0.38_B_0.24_ in 30–80 °C. This implies that the disordered structure co-exists with a small fraction of ordered structure in this sample, possibly due to the inhomogeneous distribution of the alkyl side chains. However, because of the too weak peaks from the ordered structure and the interference of the broad peak from the disordered structure, the ordered structure cannot be clearly assigned. As for the sample C18_0.23_C8_0.49_B_0.28_ with the lowest C18 content, it exhibits a broad scattering peak in its SAXS profile and thus is disordered in the whole temperature range studied. As we can see, with decreasing C18 content, the temperature-dependent structural transitions of C18*_x_*C8*_y_*B*_z_* mixed-side-chain copolymers changes from the LDOT to ordered structure in whole temperature range (including OOT), then into disordered structure in whole temperature range. In addition, the phase structure also varies with the composition of C18*_x_*C8*_y_*B*_z_* mixed-side-chain copolymers. The increase in C8 content leads to the transformation of ordered LAM structure into ordered HEX structure, then into F-K A15 phase and further into disordered structure.

Such an evolution of phase structure can be explained in terms of the changes in the volume fraction of side chains and the segregation strength between the side chains and main chain. As the C8 content increases, the volume fraction of the side chains becomes smaller, and thus, the LAM structure switches into a HEX structure. As compared with octadecyl side chain, the shorter octyl side chain and un-quaternized bromoethyl have relatively weaker segregation strengths with the main chain, resulting in a disordered structure at high C8 contents.

**Figure 2:**
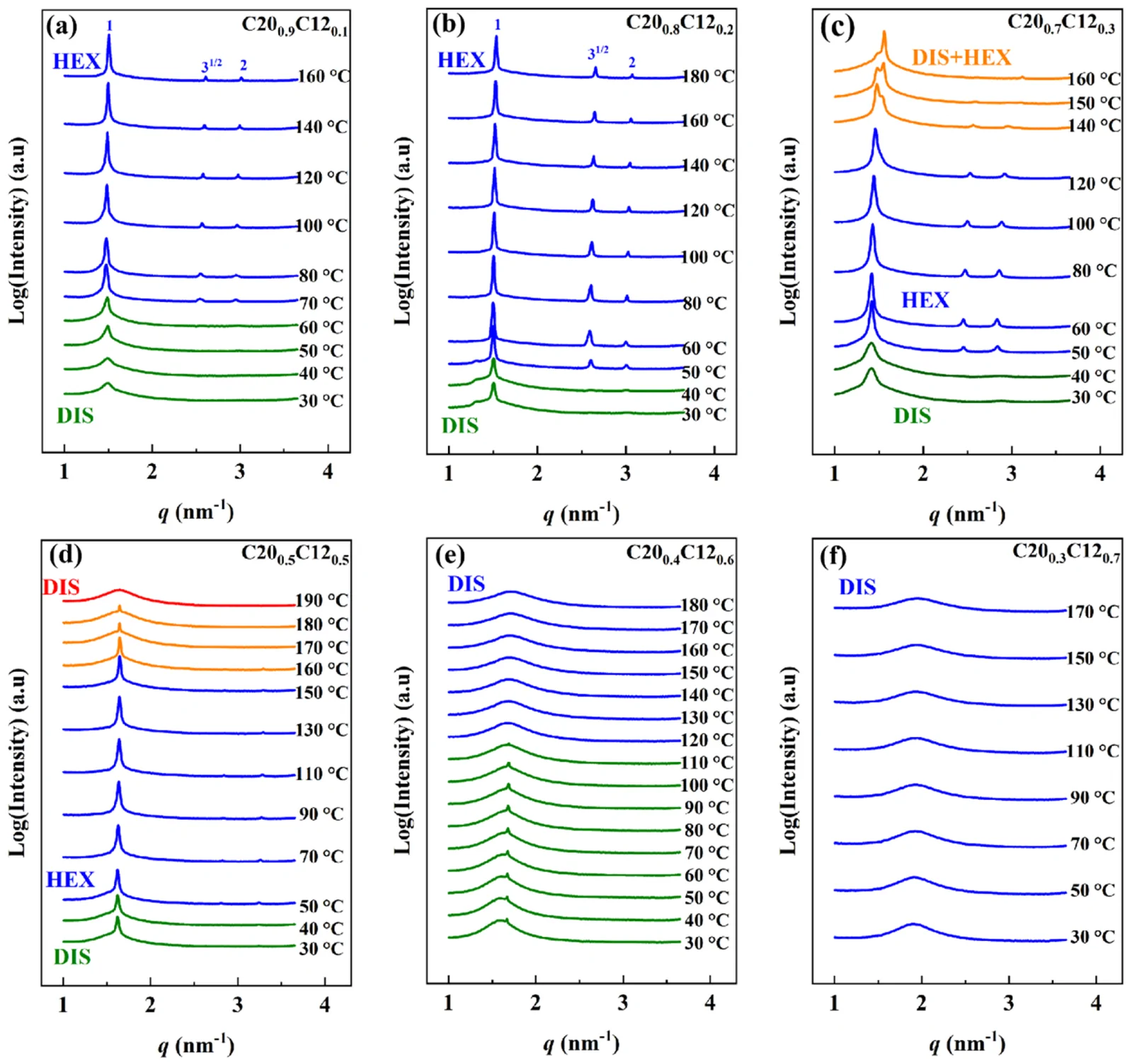
Temperature-variable SAXS profiles of (**a**) C20_0.9_C12_0.1_, (**b**) C20_0.8_C12_0.2_, (**c**) C20_0.7_C12_0.3_, (**d**) C20_0.5_C12_0.5_, (**e**) C20_0.4_C12_0.6_, (**f**) C20_0.3_C12_0.7_.

The phase behaviors of C20*_r_*C12_1-_*_r_* and C22*_r_*C12_1-_*_r_* mixed-side-chain copolymers were also investigated. Note that since the real compositions in these two series of copolymers cannot be determined, the phase behavior and structural evolution are evaluated qualitatively/semi-quantitatively with respect to the nominal feed ratio (*r*) of the monomer with longer alkyl side chain. The temperature-variable SAXS profiles of C20*_r_*C12_1-_*_r_* mixed-side-chain copolymers are illustrated in Figure 2. Like C18_0.78_C8_0.15_B_0.07_ and C18_0.60_C8_0.12_B_0.28_, the samples C20_0.9_C12_0.1_ and C20_0.8_C12_0.2_ form a disordered structure at lower temperatures, while they form an ordered HEX structure at higher temperatures, exhibiting an LDOT (Figure 2a,b). The LDOT is also observed for C20_0.7_C12_0.3_ and C20_0.5_C12_0.5_. Nevertheless, the ordered HEX structure will be partially destroyed when further heated to higher temperatures, leading to co-existence of HEX and disordered structure (Figure 2c), which may originate from the inhomogeneous distribution of two kinds of side chain along the polymer chain. In C20_0.5_C12_0.5_, the HEX structure can even be completely disrupted at 190 °C (Figure 2d), corresponding to an upper order–disorder transition (UODT). As a result, the samples C20_0.7_C12_0.3_ and C20_0.5_C12_0.5_ exhibit both an LDOT at lower temperatures and a UODT at higher temperatures, which is the characteristic of closed-loop phase transition. When the feed ratio of C20 is further decreased to 0.4 (i.e., C20_0.4_C12_0.6_), a mixture of ordered and disordered structures is formed in 30–100 °C, but it becomes totally disordered above 100 °C (Figure 2e and Figure S26). While the feed ratio of C20 is further decreased to 0.3 (i.e., C20_0.3_C12_0.7_), a totally disordered structure is formed in 30–170 °C (Figure 2f). Overall, as the feed ratio of C20 decreases, the transition sequences observed upon heating in the C20*_r_*C12_1-_*_r_* series evolves from the LDOT through closed-loop (LDOT + UODT) to disordered structures. This trend is consistent with that observed in the C18*_x_*C8*_y_*B*_z_* series.

The temperature-variable SAXS profiles of the C22*_r_*C12_1-_*_r_* series of samples are collected in Figure S30 of Supplementary Materials (and some individual SAXS profiles at selected temperatures are presented in Figures S31–S39). The sample with *r* = 0.9 exhibits mixed phase behaviors due to the heterogeneity of the chain structure: long-chain-rich segments form a stable LAM structure throughout the temperature window, while long-chain-poor segments undergo a sequential structural transition of DIS → HEX → DIS upon heating, exhibiting a closed-loop transition behavior. The samples with *r* = 0.8–0.6 also display a temperature-dependent structural transitions upon heating (LDOT + UODT), but the temperature window of the ordered HEX phase broadens as *r* decreases. For the sample with *r* = 0.5, only the LDOT is observed, and the HEX phase remains stable up to 220 °C without the appearance of UODT. The samples with *r* = 0.4–0.2 form ordered HEX over the entire temperature range; however, the low-temperature HEX structure of the sample with *r* = 0.4 is somewhat imperfect, though still identifiable as a HEX phase. For the sample with the lowest *r* = 0.1, a HEX structure forms at low temperatures but undergoes UODT at elevated temperatures, and the overall ordering degree is relatively low.

**Figure 3:**
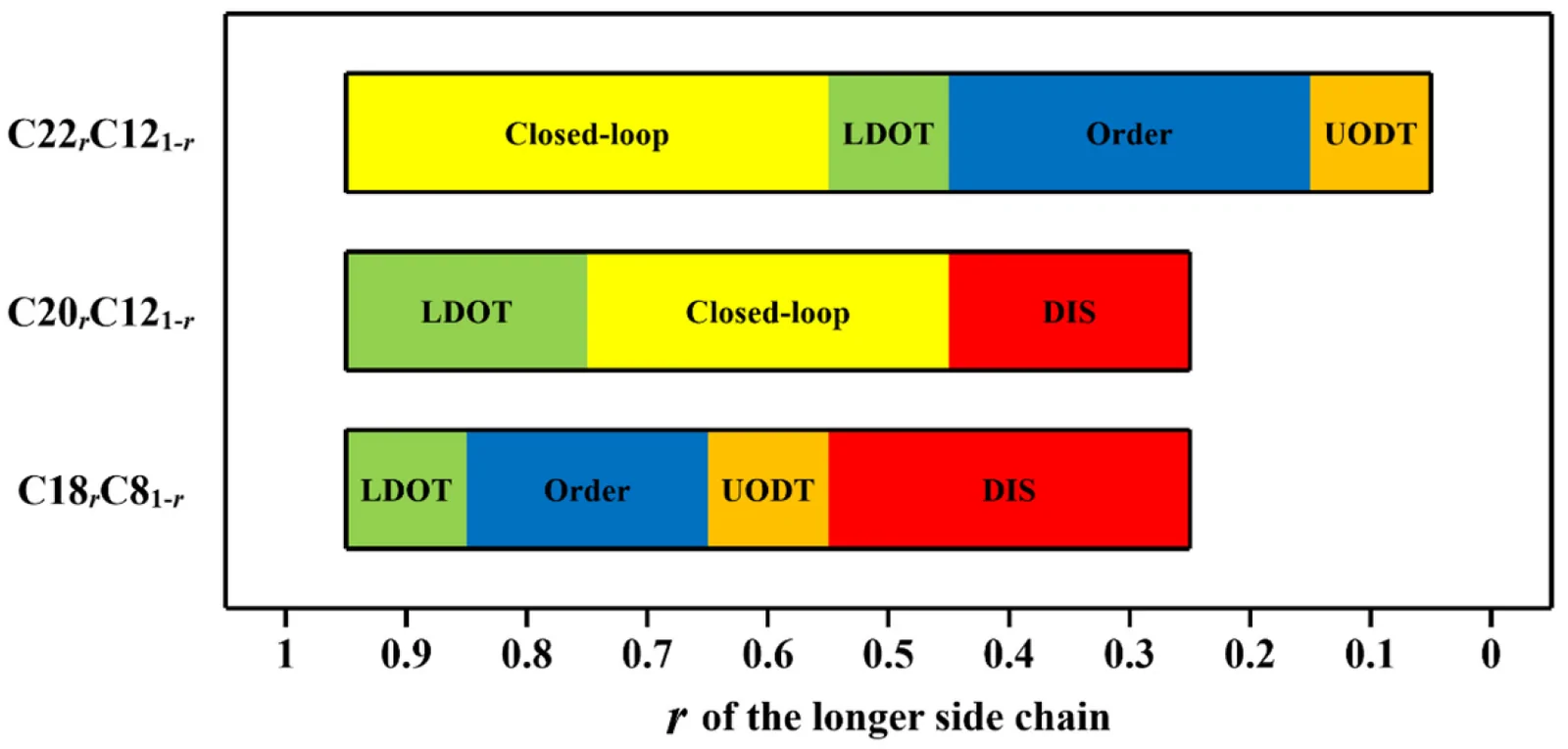
Evolution of temperature-dependent structural transitions for C18*_r_*C8_1-_*_r_*_,_ C20*_r_*C12_1-_*_r_*, and C22*_r_*C12_1-_*_r_* mixed-side-chain comb-like copolymers with the nominal feed ratio (*r*) of the longer side chain.

The evolution of temperature-dependent structural transitions for C18*_r_*C8_1-_*_r_*_,_ C20*_r_*C12_1-_*_r_*, and C22*_r_*C12_1-_*_r_* mixed-side-chain comb-like copolymers with the nominal feed ratio (*r*) of the longer side chain is summarized in Figure 3. It is observed that in the high-*r* region, the LDOT becomes more pronounced as the side-chain length increases from C18 to C22, and the LDOT region persists to lower *r* values for the C22*_r_*C12_1-_*_r_* and C20*_r_*C12_1-_*_r_* series compared with the C18*_r_*C8_1-_*_r_* analogues (C22*_r_*C12_1-_*_r_*: *r* = 0.5–0.9, C20*_r_*C12_1-_*_r_*: *r* = 0.5–0.9, and C18*_r_*C8_1-_*_r_*: *r* = 0.9) (Figure 3). This observation suggests that the occurrence of the LDOT may be correlated with certain physical properties associated with the long alkyl side chains, which will be explored in detail in the following discussion.

On the other hand, the emergence of UODT or DIS upon decreasing *r* is expected, as the reduction in nominal long-chain feed ratio inevitably leads to a weakened segregation strength between the main chain and the side chains (a lower *r* means a higher proportion of short chains, which reduces the average alkyl length and thus weakens the segregation strength between the side chains and the ionic main chain). This trend is consistently observed across all three series. Notably, however, in the low-*r* region, the C22*_r_*C12_1-_*_r_* series still retains UODT at *r* = 0.1, showing a broad ordered phase window in *r*, whereas the C18*_r_*C8_1-_*_r_* and C20*_r_*C12_1-_*_r_* series have already become fully disordered. This disparity is consistent with the higher intrinsic segregation strength imparted by the longer C22 side chains as the longer chains provide a greater hydrophobic volume fraction at the same nominal feed ratio. However, minor variations in the actual degree of quaternization (*η*) among different precursors should also be recognized. For the C22*_r_*C12_1-_*_r_* series, the closed-loop region lies to the left of the pure LDOT region compared with the C20*_r_*C12_1-_*_r_* series, which means that UODT is already observed at relatively high *r* values. Beyond intrinsic thermodynamic differences, this shift may also be partly attributed to synthetic limitations. Specifically, for precursors with longer alkyl chains (e.g., C22), lower solubility and steric hindrance could potentially result in a slightly lower actual *η* relative to the *r*. A reduced effective density of long side chains would weaken the overall segregation strength, thereby facilitating the emergence of UODT at higher nominal *r* values.

In addition, the closed-loop transition is only observed in C20*_r_*C12_1-_*_r_* and C22*_r_*C12_1-_*_r_* series, but not in C18*_r_*C8_1-_*_r_*. To clarify this observation, it is necessary to first understand the origin of the LDOT behavior and the conditions under which a sample can sequentially undergo both the LDOT and UODT upon heating. These aspects will be addressed in the following sections.

In conclusion, as the feed ratio *r* varies, the C18*_r_*C8_1-_*_r_*_,_ C20*_r_*C12_1-_*_r_*, and C22*_r_*C12_1-_*_r_* series exhibit different phase behaviors in different temperature regimes, demonstrating that the self-assembly and phase transition pathways can be effectively manipulated by tuning the side-chain length and the nominal feed stoichiometry.

### 3.2. Origin of LDOT upon Heating

The above results reveal that the mixed-side-chain comb-like copolymers with higher content of the longer alkyl side chain usually exhibit an LDOT in the low temperature regime. The LDOT phase behavior is seldom observed in BCPs and comb-like polymers, which can be either entropy-driven (for example, diblock copolymers of perdeuterated polystyrene and poly(*n*-butylmethacrylate)) [25], polystyrene-*b*-poly(*n*-butyl methacrylate) homologues [26] and poly(ethylene oxide)-*b*-poly(2-vinylpyridine) [27]) or enthalpy-driven (for example, poly(*n*-hexyl norbornene)-*b*-poly(cyclohexyl norbornene) [28], poly (acrylic acid)-*b*-poly (propylene oxide)-*b*-poly (acrylic acid) [29], PEO-*b*-poly(1-((2 acryloyloxy) ethyl)-3-methyl imidazolium) with counterions of X [30] and LiClO_4_-doped PEO-*b*-poly(*tert*-butyl acrylate-*co*-acrylic acid) [31]). However, in this work, neither of above two factors is responsible for the LDOT of mixed-side-chain comb-like copolymers. It was reported that crystallization of poly(L-lactide) (PLLA) in PLLA-containing BCPs could disrupt the pre-formed ordered structure in the melt, leading to a less ordered or even disordered structure [32,33]. In our previous work, it was found that in the BCPs composed of poly(ethylene oxide) and comb-like polyacrylate blocks, crystallization of the long alkyl side chain in the comb-like polyacrylate block also resulted in a disordered structure [34]. When the crystals were melted, the ordered structures recovered, and thus, the LDOT was observed. Herein, we suspect that the LDOT of mixed-side-chain comb-like copolymers also arises from crystallization of the alkyl side chains.

**Figure 4:**
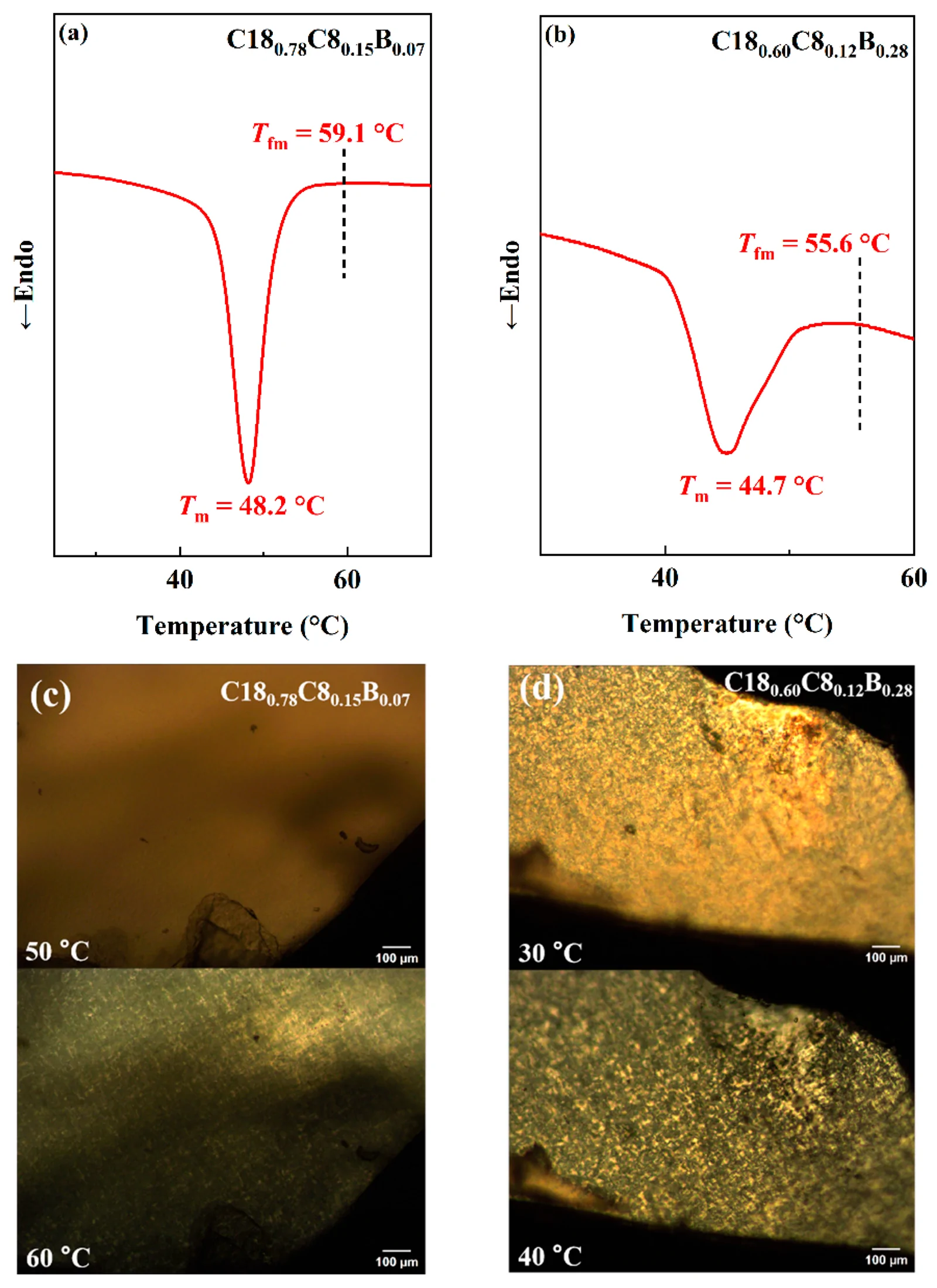
The DSC curves of (**a**) C18_0.78_C8_0.15_B_0.07_ and (**b**) C18_0.60_C8_0.12_B_0.28_. The POM images of (**c**) C18_0.78_C8_0.15_B_0.07_ at 50 °C and 60 °C and (**d**) C18_0.60_C8_0.12_B_0.28_ at 30 °C and 40 °C. The scale bars in the figures are 100 μm.

To confirm this speculation, the crystallization behavior of these samples was characterized with DSC and POM. Figure 4 shows the DSC curves and POM micrographs before and after melting of C18_0.78_C8_0.15_B_0.07_ and C18_0.60_C8_0.12_B_0.28_. It is found that C18_0.78_C8_0.15_B_0.07_ exhibits an endothermic melting peak with the melting temperature (*T*_m_) centered at ~48.2 °C, with a final melting temperature (*T*_fm_) of ~59.1 °C (Figure 4a). Bright birefringent patterns are observed in its POM micrographs at 50 °C (Figure 4c), confirming the presence of anisotropic crystalline structures. However, these patterns appear as a uniform bright background without discernible texture, suggesting that the crystallites are randomly oriented. Upon heating to 60 °C, the field of vision in POM becomes slightly darker (Figure 4c), but simultaneously, alternating bright-and-dark textures emerge, indicating the formation of a liquid crystalline phase. This temperature coincides approximately with the temperature at which the ordered LAM structure appears (~60 °C), determined by SAXS (Figure 1a and Figure 4b present the DSC curve of C18_0.60_C8_0.12_B_0.28_, in which a broad endothermic melting peak is observed with the *T*_m_ = 44.7 °C and *T*_fm_ = 55.6 °C, suggesting weaker crystallinity of this sample compared to C18_0.78_C8_0.15_B_0.07_). Additionally, C18_0.60_C8_0.12_B_0.28_ displays bright birefringent patterns at 30 °C, indicating the presence of anisotropic ordered structures (possibly local crystallization); upon heating to 40 °C, the field of view exhibited clear extinction (Figure 4d), and the extinction temperature again coincided with the *T*_DOT_ (~40 °C) determined by SAXS (Figure 1b). Notably, in this sample with lower C18 side-chain content, the texture remains relatively clear even at lower temperatures (e.g., 30 °C), suggesting that the reduced crystallinity allows the system to directly form a liquid crystalline phase, which further develops upon heating and gives rise to increasingly distinct alternating bright-and-dark textures. For other C18*_x_*C8*_y_*B*_z_* samples, no melting and crystallization peaks and birefringence phenomenon are detected by DSC and POM, respectively, implying the incapability of crystallization of the side chains.

**Figure 5:**
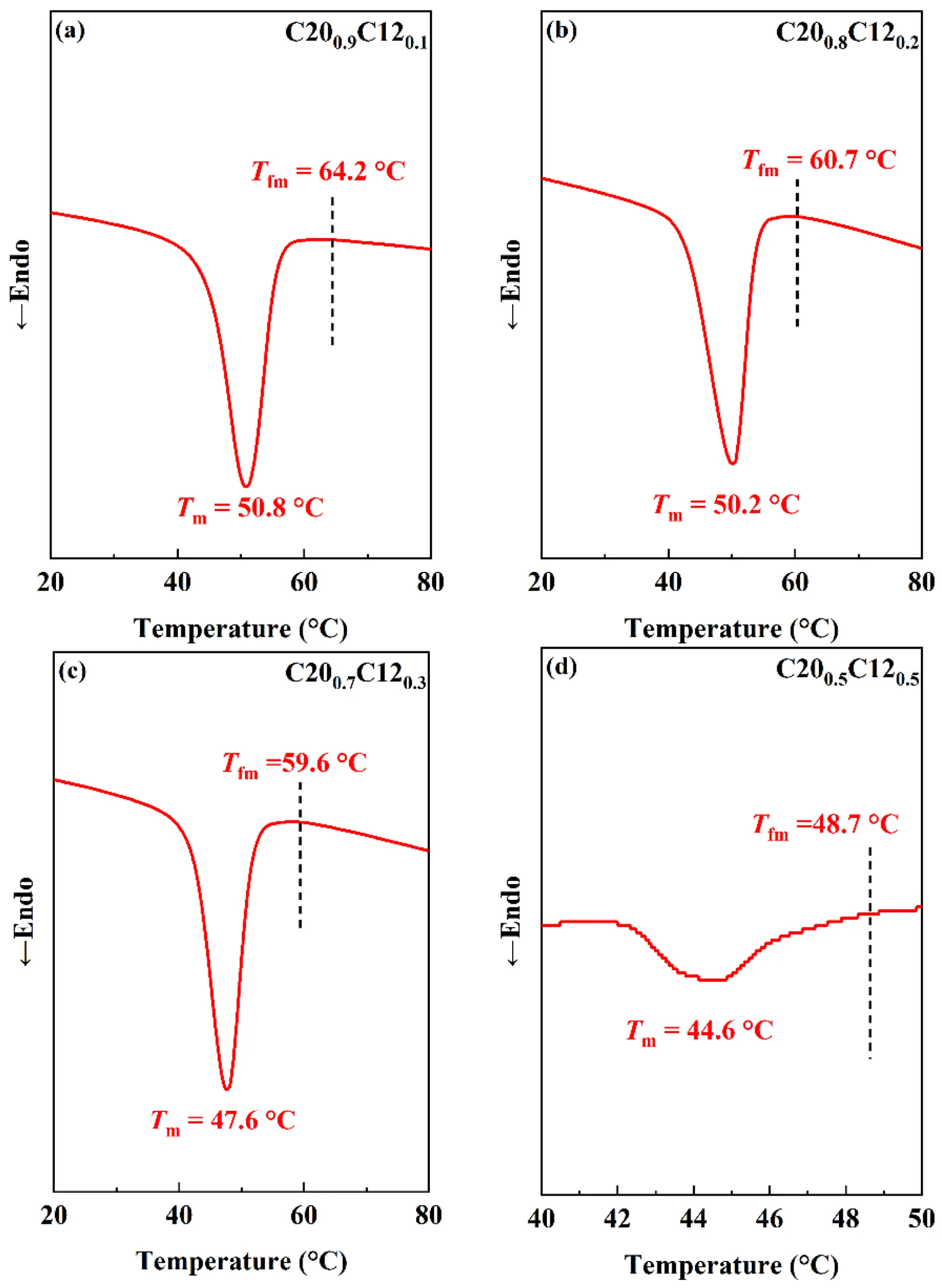
The DSC curves of (**a**) C20_0.9_C12_0.1_, (**b**) C20_0.8_C12_0.2_, (**c**) C20_0.7_C12_0.3_, and (**d**) C20_0.5_C12_0.5_.

Figure 5 shows the DSC curves of C20_0.9_C12_0.1_, C20_0.8_C12_0.2_, C20_0.7_C12_0.3_ and C20_0.5_C12_0.5_. For C20_0.9_C12_0.1_, the DSC curve exhibits a melting peak with a *T*_fm_ of 64.2 °C (Figure 5a), which coincides approximately with the *T*_DOT_ (~70 °C) determined by SAXS (Figure 2a). For C20_0.8_C12_0.2_, a melting peak is also observed with *T*_m_ = 50.2 and *T*_fm_ = 60.7 °C (Figure 5b), again approximately matching its *T*_DOT_ (~50 °C) (Figure 2b). In contrast, C20_0.7_C12_0.3_ shows lower *T*_m_ (47.6 °C) and *T*_fm_ (59.6 °C) (Figure 5c), approximately matching *T*_DOT_ (~50 °C) (Figure 2c). C20_0.5_C12_0.5_ shows *T*_m_ (44.6 °C) and *T*_fm_ (48.7 °C) (Figure 5d), approximately matching *T*_DOT_ (~50 °C) (Figure 2d). The DSC curves of the C22*_r_*C12_1-_*_r_* series are shown in Figure S41. It was found that when the C22 feed fraction *r* is ≥ 0.4, the samples are crystallizable, and the *T*_fm_ is close to *T*_DOT_. The phase transition temperatures determined from SAXS and DSC are summarized in Table 1. It is worth noting that owing to the 10 °C temperature increment used in the SAXS measurements, the actual *T*_DOT_ may exhibit minor discrepancies relative to the melting temperatures derived from DSC. For other samples, no crystallization or melting peaks are detected, indicating the absence of side-chain crystallinity.

The DSC and POM results show that crystallization of the longer alkyl side chains in the mixed-side-chain comb-like copolymers can indeed be correlated with the LDOT phase behavior of them. Crystallization of the alkyl side chain will disrupt the ordered structure pre-formed in the melt, leading to a disordered structure at lower temperatures, which is solidified by crystallization. As a result, the LDOT phase behavior is observed. When the contents of two kinds of alkyl side chains are comparable, they cannot crystallize due to mutual interference, and the LDOT phase behavior disappears accordingly. However, it should be noted that the P(AOEA*_m_*I-Br) homopolymers do not exhibit a disorder–order transition upon melting of the side chains [22]. This is because the homopolymers with uniform alkyl side chains have stronger crystallizability; thus, uniform crystalline structure is formed, and the ordered LAM structures can be retained after crystallization. This also reveals that the phase behavior of comb-like copolymers can be effectively tuned by mixed-side-chain functionalization.

**Figure 6:**
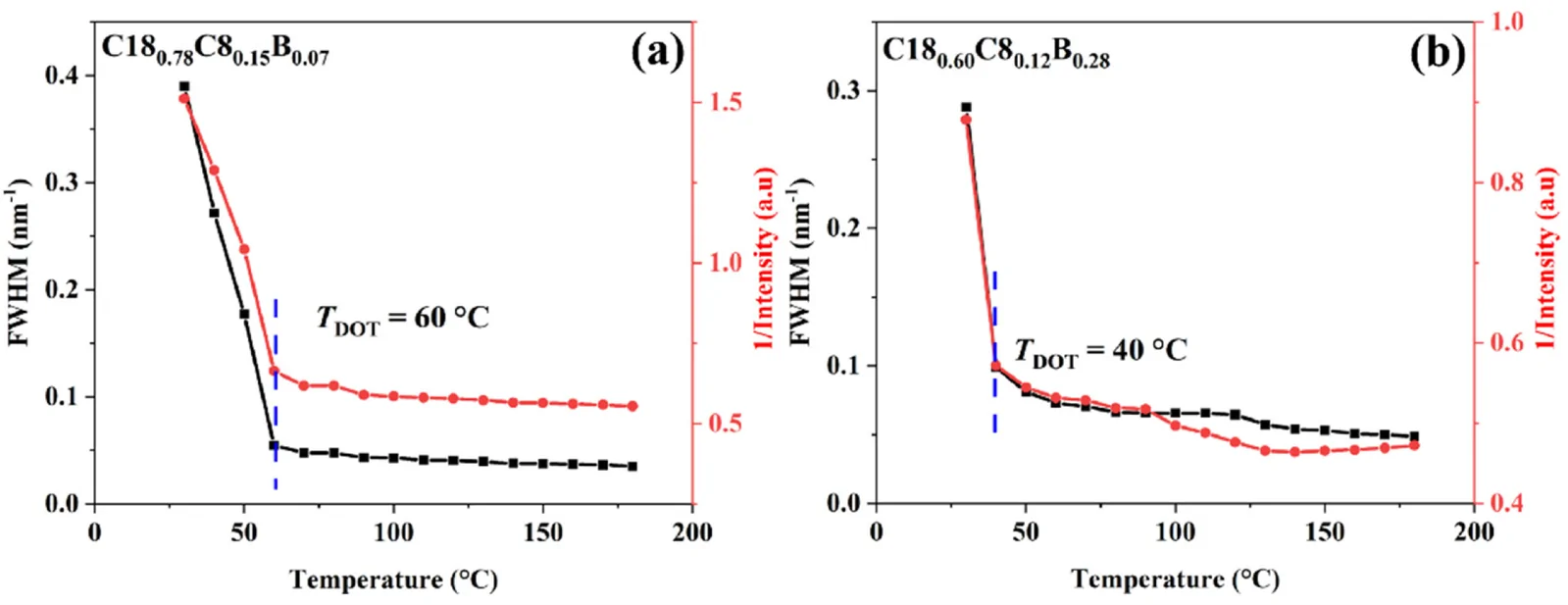
The FWHM (black squares, left axis) and 1/Intensity (red circles, right axis) of the SAXS primary peaks at various temperatures for (**a**) C18_0.78_C8_0.15_B_0.07_ and (**b**) C18_0.60_C8_0.12_B_0.28_ upon heating. The blue dotted lines show the possible phase transition temperatures.

When phase transition occurs in BCP, the FWHM and intensity of the primary scattering peak will change drastically; thus, the FWHM and reciprocal intensity (1/Intensity) of the primary scattering peak are usually plotted as a function of temperature to determine the phase transition temperatures, such as *T*_ODT_ and *T*_DOT_ [24]. Figure 6 shows the plots of the FWHM ~*T* and 1/Intensity ~*T* for C18_0.78_C8_0.15_B_0.07_ and C18_0.60_C8_0.12_B_0.28_. One can see from Figure 6 that both the FWHM and 1/Intensity decrease abruptly at ~60 °C and ~40 °C for C18_0.78_C8_0.15_B_0.07_ and C18_0.60_C8_0.12_B_0.28_, respectively, which can be viewed as the *T*_DOT_ of them.

It is worth noting that while most samples in the C18*_x_*C8*_y_*B*_z_* series were synthesized using the P4 precursor (*M*_n_ = 68.7 kg/mol, *η* ≈ 72–76%), the sample with the highest C18 content (C18_0.78_C8_0.15_B_0.07_) was prepared from the P1 precursor (*M*_n_ = 34.1 kg/mol) with a higher *η* ≈ 93%. Consequently, the lower backbone molecular weight and higher degree of quaternization in this sample act as confounding variables alongside the nominal feed ratio. Higher *η* increases the electrostatic and dipolar interactions, which might synergize with the high C18 fraction to facilitate side-chain crystallization and the manifestation of LDOT behavior. Although the long-chain content remains the primary driving force for side-chain ordering, the potential contributions of *M*_n_, *η*, and *r* cannot be fully decoupled for this specific composition, and this limitation should be considered when comparing the phase behaviors across different compositions.

**Figure 7:**
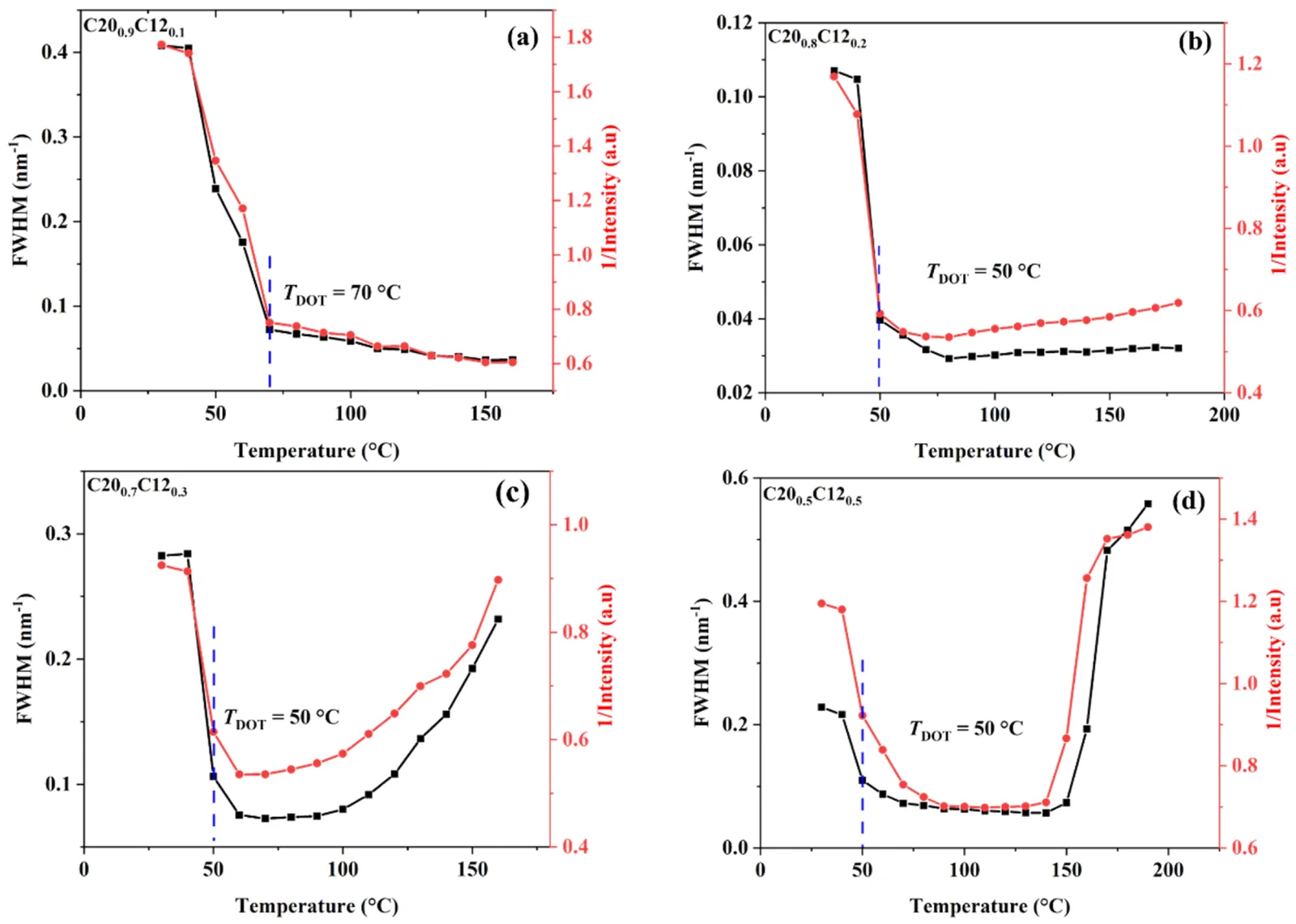
The FWHM (black squares, left axis) and 1/Intensity (red circles, right axis) of the SAXS primary peaks at various temperatures upon heating for (**a**) C20_0.9_C12_0.1_, (**b**) C20_0.8_C12_0.2_, (**c**) C20_0.7_C12_0.3_, and (**d**) C20_0.5_C12_0.5_. The blue dotted lines show the possible phase transition temperatures.

Figure 7 shows the plots of the FWHM ~*T* and 1/Intensity ~*T* for the C20*_r_*C12_1-_*_r_* series samples. For C20_0.9_C12_0.1_ and C20_0.8_C12_0.2_, both the FWHM and 1/Intensity exhibit an abrupt decrease at ~70 °C and ~50 °C, respectively, which are taken as their *T*_DOT_ (also the corner point temperatures of the curves) (Figure 7a,b). For C20_0.7_C12_0.3_ and C20_0.5_C12_0.5_, a decrease in the FWHM and 1/Intensity at low temperatures is observed, followed by an increase at higher temperatures, indicating the simultaneous occurrences of the LDOT and UODT, i.e., closed-loop phase behavior. However, the corner point temperatures are not taken as the *T*_DOT_ or *T*_ODT_ for these two samples_._ This is mainly because compositional heterogeneity along the polymer backbones leads to the coexistence of ordered and disordered phases over a broad temperature range. Well-defined higher-order scattering peaks (e.g., √3q* and 2q*) already appear before the first corner points (Figures S27 and S28), and the UODT occurs at ~140 °C and ~170 °C, respectively (Figure 7c,d). For C20_0.4_C12_0.6_, the system is already in a fully disordered state at low temperatures, as evidenced by the absence of higher-order scatterings in its SAXS profiles (Figure S29). This behavior is consistent with the expected response of a system that lacks sufficient segregation strength to form an ordered structure across the entire temperature range. The plots of the FWHM ~*T* and 1/Intensity ~ *T* for the C22*_r_*C12_1-_*_r_* series samples are provided in the Supplementary Materials (Figure S40).

It is noticed that the *T*_DOT_ decreases with increasing the content of the shorter alkyl side chain. Therefore, the *T*_DOT_ of mixed-side-chain comb-like copolymers can be tuned by the composition of the side chain.

Combining these results, we can infer that the crystallization of long alkyl side chains is primarily responsible for the disordered state at low temperatures. The long alkyl side chains tend to crystallize imperfectly at low temperatures under the interference of short alkyl side chains, which will disrupt the pre-formed microphase-separated structure, rendering the system macroscopically disordered or weakly ordered—a phenomenon referred to as “break-out crystallization,” which has also been reported in crystalline-rubbery BCPs [35,36,37]. Upon heating above the side-chain melting temperature, the crystalline structure of the alkyl side chains melts, eliminating the perturbation to microphase separation, and the system rearranges under the driving force of main-chain/side-chain incompatibility, recovering the ordered nanostructure. In summary, the above results strongly suggest that side-chain crystallization/melting plays a major role in the observed LDOT behavior.

### 3.3. Frank–Kasper A15 Phase

**Figure 8:**
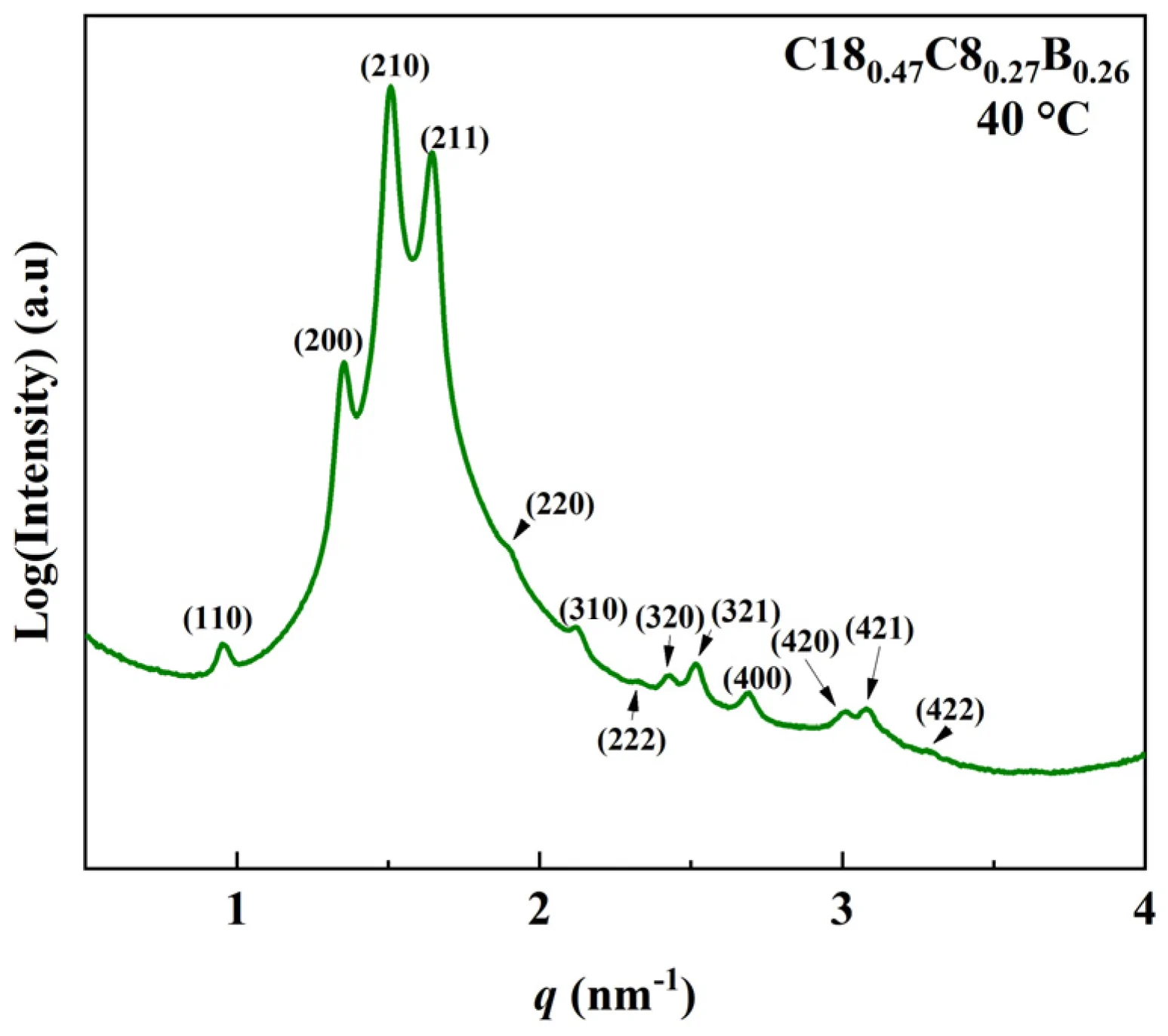
SAXS profile of C18_0.47_C8_0.27_B_0.26_ at 40 °C.

Figure 1d shows that the SAXS profiles of C18_0.47_C8_0.27_B_0.26_ at low temperatures do not exhibit the characteristics of common LAM, HEX, gyroid (GYR), and body-centered cube (BCC) structures, indicating a more complex phase structure. For better assignment of such a structure, the enlarged SAXS profile of C18_0.47_C8_0.27_B_0.26_ at 40 °C is presented in Figure 8, in which more than ten reflections, together with three sharp peaks ((200), (210), and (211)) in the low-*q* region preceded by a (110) weaker peak, are observed. This is the characteristic of Frank–Kasper (F-K) A15 phase [23]. All reflections can be fully indexed according to the cubic *Pm*$\bar{3}$*n* space group (Table S5). The unit cell parameter *a* calculated based on the (110) reflection is 9.35 nm, and the residuals between the experimental scattering vectors *q*_obs_ and the calculated values *q*_calc_ are all below 0.76%.

To further validate the A15 assignment, we compared our SAXS profile with the A15 phase reported by Lindsay et al. in bidisperse polystyrene-*b*-polybutadiene diblock copolymer blends [23]. The higher-order reflections, including (220), (310), (222), (320), (321), (400), (420), (421), and (422), also match well with those observed in the literature. No extraneous peaks from potential competing phases, such as BCC or *σ*, are discernible in the pattern, further supporting the phase assignment. It is noted that several reflections reported in the literature, such as (410), (411), (322), (430), (510), (520), (521), and (440), are not clearly resolved in our pattern; instead, weak diffuse features appear in the vicinity of (400) and (421). This is likely because these high-order reflections intrinsically possess low scattering intensities, making them difficult to detect in the present system, particularly given the limited contrast arising from the weakly electron-density-contrasting alkyl side chains and the ionic main chain. Nevertheless, the excellent agreement of the major reflections, the absence of peaks characteristic of other structures, and the low residuals between *q*_obs_ and *q*_calc_ (<0.76%) collectively provide evidence that the SAXS data are consistent with the A15 phase.

The F-K A15 phase has attracted considerable attention in soft matter self-assembly due to its unique lattice structure. The neat linear diblock copolymers are difficult to form F-K A15 phase directly. However, the F-K A15 phase can be obtained via blending linear BCPs with different molecular weights and compositions or using BCPs with branching topological chain structures [23,38]. Le et al. [39] first reported the F-K A15 phase in a random graft copolymer with PDMS and PEO as the side chains. Although the mixed-side-chain comb-like copolymers in this work belong to the same broad category of “side-chain graft” copolymers as the system of Le et al., there are significant differences in molecular design and driving force for self-assembly. In Le’s system, the formation of the A15 phase is primarily driven by the chemical incompatibility between PDMS and PEO side chains, falling within the classical realm of side-chain/side-chain microphase separation. In contrast, in our system, the driving force for phase separation originates from the incompatibility between the main chain (containing imidazolium ionic junctions) and the alkyl side chains. Moreover, because the domain spacing is primarily determined by the side-chain length, the unit cell parameter of the A15 phase in our system is only *a* = 9.35 nm, significantly smaller than that reported by Le et al. (approximately 15.5 nm) and substantially smaller than the typical dimensions of F-K phases in conventional BCPs (~100 nm).

It should be pointed out that the F-K A15 phase was not observed in the P(AOEA*_m_*I-Br) homopolymers. It is also worth noting that the sample C18_0.47_C8_0.27_B_0.26_ contains approximately 26% non-quaternized BrEA repeat units (B*_z_* fraction). Therefore, this system is not a simple binary mixture of C18- and C8-functionalized side chains but rather a ternary system comprising C18-functionalized, C8-functionalized, and unfunctionalized bromoethyl units. The presence of these neutral bromoethyl units may play a non-negligible role in stabilizing the high-curvature A15 phase, as they introduce additional compositional asymmetry and local variations in segregation strength along the polymer backbone. This additional compositional complexity, which is absent in the P(AOEA*_m_*I-Br) homopolymers, may be a key factor enabling the formation of the A15 phase in this particular sample. Thus, while the mixing of two alkyl side-chain lengths is essential for tuning the effective volume fraction and interfacial curvature, the A15 formation should be understood as arising from the combined effects of side-chain composition distribution and the presence of unquaternized units rather than solely from the binary mixing of side chains.

### 3.4. Temperature Dependence and Tunability of D-Spacing

**Figure 9:**
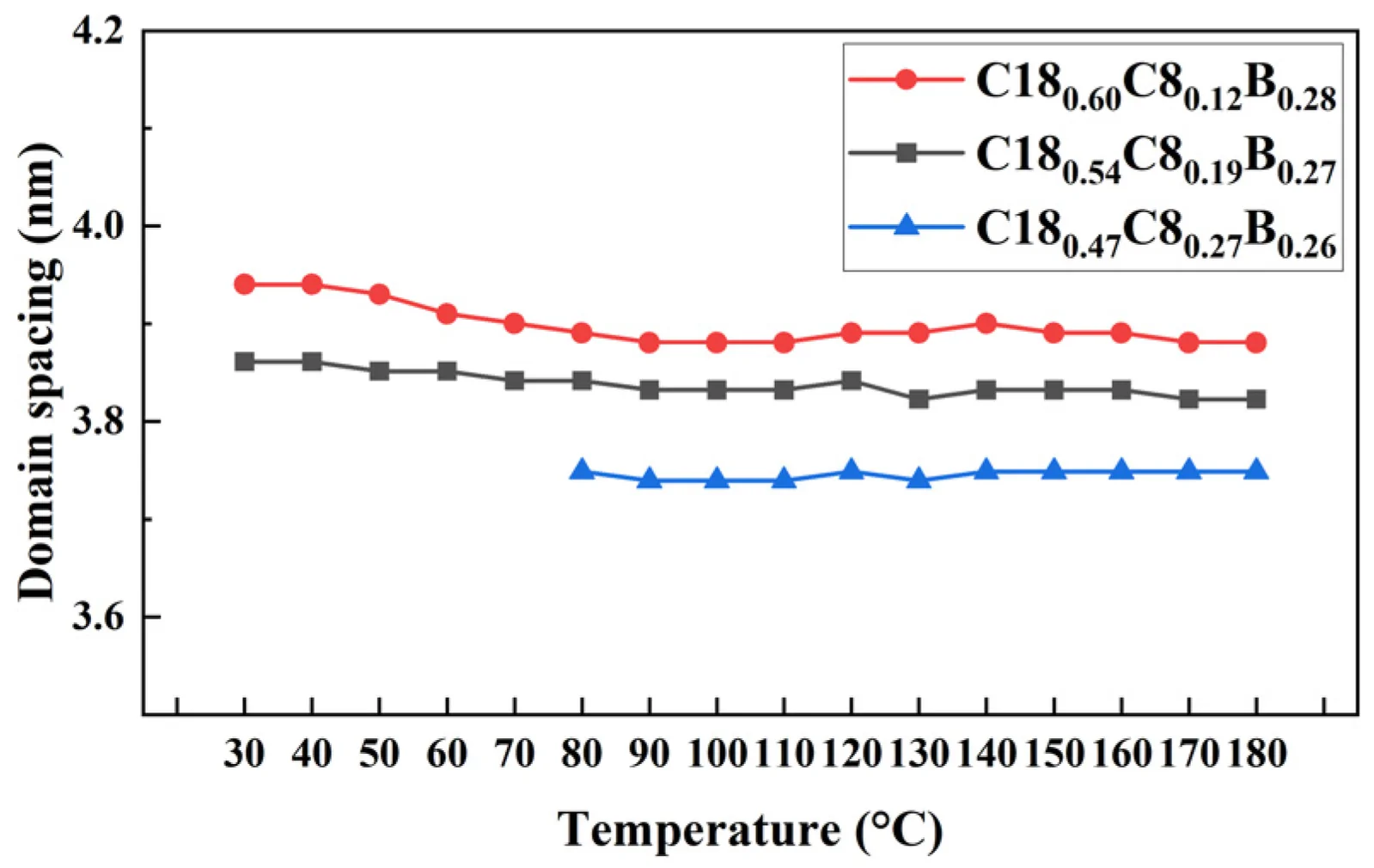
Domain spacings of the HEX phase for C18*_x_*C8*_y_*B*_z_* as a function of temperature.

The domain spacing (*d*) of the phase structures can be calculated from scattering vector of the primary peak (q*) using the equation *d* = 2π/q*. Figure 9 shows the *d* values of three C18*_x_*C8*_y_*B*_z_* samples with a HEX structure at different temperatures. It is observed that the *d* values of all three samples vary very slightly with temperature since the fluctuation of *d* is within 0.1 nm. It should be noted that C18_0.47_C8_0.27_B_0.26_ does not form a HEX phase in 30–70 °C, and therefore, the data of C18_0.47_C8_0.27_B_0.26_ in this temperature range are not included for comparison. This demonstrates that the HEX domain spacing shows low thermal expansion within the investigated temperature window.

**Figure 10:**
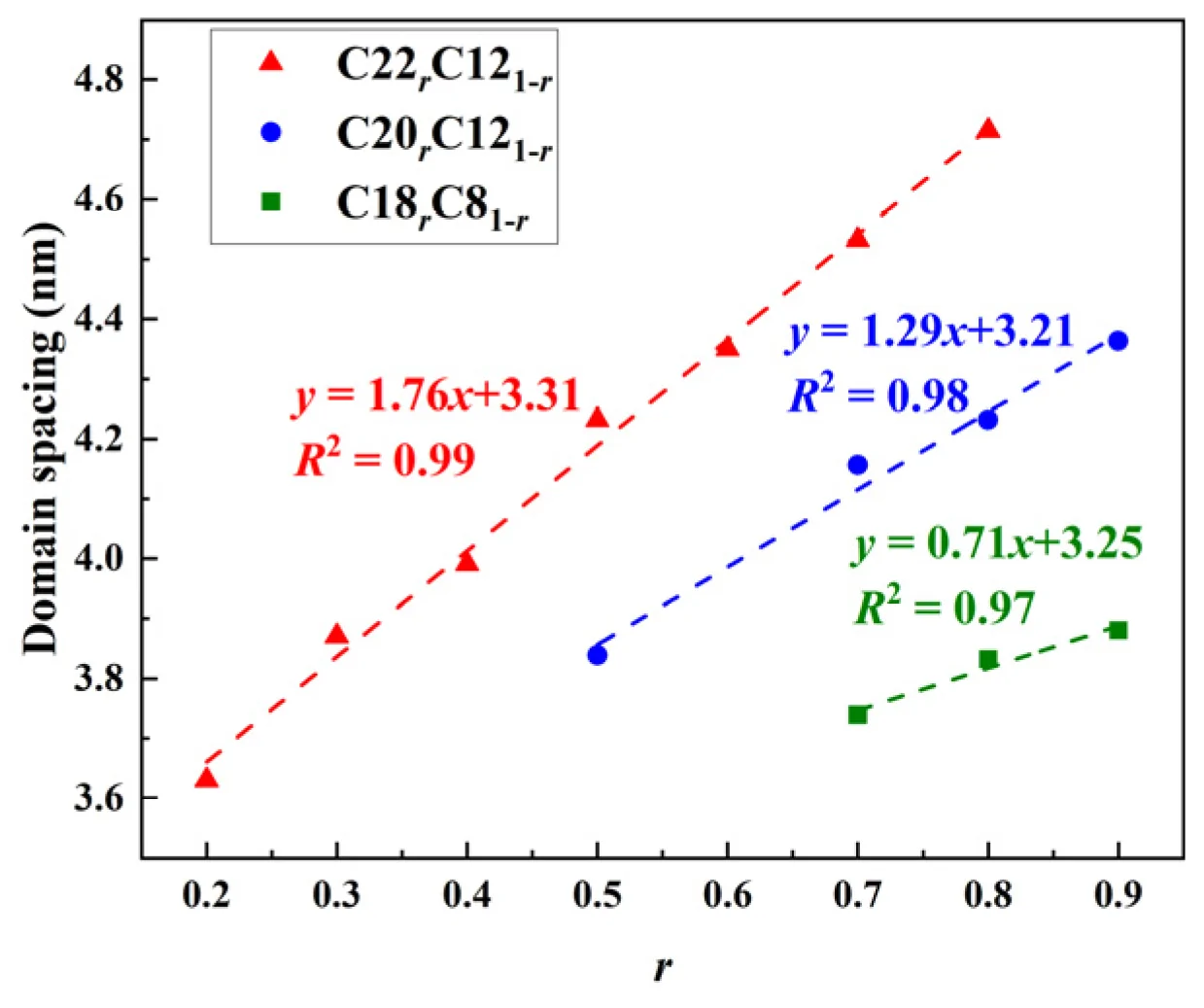
Domain spacings of the HEX phase for C22*_r_*C12_1-*r*_ (red triangles), C20*_r_*C12_1-*r*_ (blue circles), and C18*_r_*C8_1-*r*_ (green squares) as a function of nominal feed ratio.

To investigate the structural dimensions, we systematically investigated the evolution of *d* by adjusting the nominal feed ratio *r* of the monomer with longer side chain. For the three series of mixed-side-chain comb-like copolyacrylates with different alkyl side-chain lengths (C22/C12, C20/C12, C18/C8), the domain spacings of the HEX phase at 100 °C are plotted as a function of the nominal feed ratio (Figure 10). First, for all three series of samples, the domain spacing of the HEX phase shows an approximately linear increase with increasing the nominal feed ratio (*R*^2^ = 0.98). These results suggest that the domain spacing exhibits an approximately linear dependence on the nominal long-chain feed ratio within the investigated composition range. Note that only three data points with *r* = 0.7, 0.8 and 0.9 are used for the C18*_r_*C8_1-*r*_ samples because the C18*_r_*C8_1-_*r* samples can form an ordered HEX structure only at relatively high feed ratios. Furthermore, the slopes of the three fitting lines increase from 0.71 to 1.76 gradually as the longer side-chain unit changes from C18 to C22. This trend indicates that longer-chain comb-like copolymers display a stronger apparent responsiveness of domain spacing with respect to the nominal feed ratio. This behavior may reflect potential differences in chain packing, steric hindrance, and conformational stretching at the phase-separation interface, which could amplify the influence of nominal composition on interfacial curvature and domain spacing.

To assess the possible influence of absorbed water on the microphase-separated structures, a thermogravimetric analysis (TGA) was performed on a representative sample (C18_0.78_C8_0.15_B_0.07_). As shown in Figure S42, a gradual and continuous weight loss of approximately 2.38% was observed upon heating from room temperature to 140 °C, which is attributed to the desorption of adsorbed water and trace residual solvents. This indicates that a small amount of water was present in the vacuum-dried samples. Given the sub-5 nm domain sizes in this system, the potential effect of such residual water on the observed domain spacings and phase behavior should be noted.

## 4. Conclusions

This work demonstrates that by varying the nominal feed ratio (*r*) of long-chain alkyl side groups and the alkyl side-chain length, the microphase separation behavior of the charged mixed-side-chain comb-like copolymers can be systematically regulated. Specifically, depending on the side-chain composition, the systems can exhibit diverse temperature-dependent transition sequences upon heating, including the simple LDOT and UODT, closed-loop (LDOT + UODT), and thermally stable ordered structure without transition. Meanwhile, the phase structure can be switched among lamellar (LAM), hexagonally packed cylindrical (HEX), and complex spherical phase, including one consistent with the Frank–Kasper A15 structure. Moreover, both *T*_DOT_ and *T*_ODT_ shift with changes in the side-chain length and *r*. Furthermore, the domain spacings of the ordered structures show an approximately linear dependence on *r* and can be tuned in the range of 3–5 nm. In summary, the mixed-side-chain functionalization strategy enables multi-dimensional and synergistic regulation of the phase behavior, phase structure, phase transition temperature, and domain spacing in this type of charged comb-like copolymer, offering an effective route toward the design of ultra-small microphase-separated structures.

## Figures and Tables

**Table 1:** *T*_m_, *T*_fm_, and *T*_DOT_ for the samples with the LDOT upon heating.

Samples	*T*_m_ (°C)	*T*_fm_ (°C)	*T*_DOT_ (°C)
C18_0.78_C8_0.15_B_0.07_	48.2	59.1	~60
C18_0.60_C8_0.12_B_0.28_	44.7	55.6	~40
C20_0.9_C12_0.1_	50.8	64.2	~70
C20_0.8_C12_0.2_	50.2	60.7	~50
C20_0.7_C12_0.3_	47.6	59.6	~50
C20_0.5_C12_0.5_	44.6	48.7	~50
C22_0.9_C12_0.1_	58.5	72.3	~70
C22_0.8_C12_0.2_	55.4	70.5	~60
C22_0.7_C12_0.3_	53.4	64.5	~60
C22_0.6_C12_0.4_	51.1	62.4	~50
C22_0.5_C12_0.5_	48.8	60.3	~50
C22_0.4_C12_0.6_	47.1	54.4	~50

## Data Availability

The original contributions presented in the study are included in the article/Supplementary Materials, further inquiries can be directed to the corresponding authors.

## Supplementary Materials

The following supporting information can be downloaded at https://www.mdpi.com/article/10.3390/ma19183955/s1. Figure S1: ^1^H-NMR spectrum of BrEA; Figure S2: GPC traces of PBrEA samples; Table S1: Molecular characteristics and polymerization conditions of PBrEA; Figure S3: ^1^H-NMR spectrum of PBrEA sample P4; Figure S4: ^1^H-NMR spectrum of 1-dodecylimidazole; Figure S5: ^1^H-NMR spectrum of 1-octadecylimidazole; Figure S6: ^1^H-NMR spectrum of 1-eicosylimidazole; Figure S7: ^1^H-NMR spectrum of 1-docosylimidazole; Table S2: Main chemical shifts of individual homologues; Figure S8: ^1^H-NMR spectra (expanded methylene region) of the 1-alkylimidazole series (C12–C22); Figure S9: ^13^C-NMR spectrum of 1-octadecylimidazole; Figure S10: ^13^C-NMR spectrum of 1-eicosylimidazole; Figure S11: ^13^C-NMR spectrum of 1-docosylimidazole; Figure S12: ^1^H-NMR spectrum of C18_0.60_C8_0.12_B_0.28_; Table S3: Molecular characteristics of C18*_x_*C8*_y_*B*_z_*; Figure S13: ^1^H-NMR spectrum of C18_0.78_C8_0.15_B_0.07_; Figure S14: ^1^H-NMR spectrum of C18_0.54_C8_0.19_B_0.27_; Figure S15: ^1^H-NMR spectrum of C18_0.47_C8_0.27_B_0.26_; Figure S16: ^1^H-NMR spectrum of C18_0.38_C8_0.38_B_0.24_; Figure S17: ^1^H-NMR spectrum of C18_0.30_C8_0.43_B_0.27_; Figure S18: ^1^H-NMR spectrum of C18_0.23_C8_0.49_B_0.28_; Figure S19: ^1^H-NMR spectrum of C18_0.20_C8_0.53_B_0.27_; Figure S20: Solubility of C22_0.5_C12_0.5_ in DMSO solvent; Figure S21: ^1^H-NMR spectrum of C22_0.5_C12_0.5_; Figure S22: SAXS profile of C18_0.78_C8_0.15_B_0.07_ at 80 °C; Figure S23: SAXS profile of C18_0.60_C8_0.12_B_0.28_ at 80 °C; Figure S24: SAXS profile of C18_0.54_C8_0.19_B_0.27_ at 80 °C; Table S4: Representative Lorentz peak fitting parameters for the primary SAXS peak of C18_0.78_C8_0.15_B_0.07_; Table S5: Assignments of the scattering peaks for C18_0.47_C8_0.27_B_0.26_; Figure S25: SAXS profile of C20_0.9_C12_0.1_ at 70 °C; Figure S26: SAXS profiles of C20_0.8_C12_0.2_ at (a) 30 °C. and (b) 70 °C; Figure S27: SAXS profiles of C20_0.7_C12_0.3_ at (a) 50 °C and (b) 160 °C; Figure S28: SAXS profiles of C20_0.5_C12_0.5_ at (a) 50 °C, (b) 170 °C and (c) 190 °C; Figure S29: SAXS profile of C20_0.4_C12_0.6_ at 60 °C; Figure S30: Temperature-variable SAXS profiles of (a) C22_0.9_C12_0.1_, (b) C22_0.8_C12_0.2_, (c) C22_0.7_C12_0.3_, (d) C22_0.6_C12_0.4_, (e) C22_0.5_C12_0.5_, (f) C22_0.4_C12_0.6_, (g) C22_0.3_C12_0.7_, (h) C22_0.2_C12_0.8_, and (i) C22_0.1_C12_0.9_; Figure S31: SAXS profiles of C22_0.9_C12_0.1_ at (a) 30 °C, (b) 70 °C and (c) 100 °C; Figure S32: SAXS profiles of C22_0.8_C12_0.2_ at (a) 70 °C, (b) 100 °C and (c) 200 °C; Figure S33: SAXS profiles of C22_0.7_C12_0.3_ at (a) 70 °C and (b) 200 °C; Figure S34: SAXS profiles of C22_0.6_C12_0.4_ at (a) 70 °C and (b) 200 °C; Figure S35: SAXS profiles of C22_0.5_C12_0.5_ at (a) 30 °C and (b) 70 °C; Figure S36: SAXS profiles of C22_0.4_C12_0.6_ at (a) 30 °C and (b) 70 °C; Figure S37: SAXS profile of C22_0.3_C12_0.7_ at 30 °C; Figure S38: SAXS profile of C22_0.2_C12_0.8_ at 30 °C; Figure S39: SAXS profiles of C22_0.1_C12_0.9_ at (a) 30 °C and (b) 160 °C; Figure S40: FWHM and 1/Intensity of SAXS primary peaks for C22*_r_*C12_1-_*_r_* series; Figure S41: DSC curves of C22*_r_*C12_1-_*_r_* series; Figure S42: TGA curve of C18_0.78_C8_0.15_B_0.07_.
