# Supplementary material for: Microphase Separation and Temperature-Dependent Structural Transitions of Mixed-Side-Chain Comb-like Copolymers
Source: Materials (Basel). 2026 Sep 17;19(18):3955. doi: 10.3390/ma19183955 (PMC13608407; doi:10.3390/ma19183955)
Supplement: Supplementary file 1 [file materials-19-03955-s001.zip › materials-4528349-supplementary.pdf]

Supplementary Materials

Microphase Separation and Temperature-Dependent Structural Transitions of Charged Mixed-side-chain Comb-like Copolymers

Yuan-Hao Yao [1,*], Jun-Ting Xu [2] and Ze-Kun Zhang [3,*]

1 State Key Laboratory of Biobased Transportation Fuel Technology, Department of Polymer Science and Engineering, Zhejiang University, Hangzhou 310058, China

2 State Key Laboratory of Biobased Transportation Fuel Technology, Department of Polymer Science and Engineering, Zhejiang University, Hangzhou 310058, China

3 Dalian Changfeng Industrial Corporation, Dalian 116038, China

* Correspondence: 22329008@zju.edu.cn (Y.-H.Y.); 976112814@qq.com (Z.-K.Z.)

Contents:

1. Part 1: Synthesis and structure characterization: **S2**
2. Part 2: Phase behaviors of the comb-like copolymers: **S18**
3. Part 3: DSC and TGA curves of comb-like copolymers: **S31**

Part 1: Synthesis and structure characterization

Synthesis of 2-bromoethyl acrylate (BrEA)

A mixture of 2-bromoethanol (62.48 g, 0.5 mol), EDCI (120 g, 0.625 mol), and DMAP (6.01 g, 0.05 mol) was dissolved in CH_2Cl_2 (270 mL) and stirred in an ice-water bath for 0.5 h. Acrylic acid (45.04 g, 0.625 mol) was dissolved in CH_2Cl_2 (80 mL) and then added dropwise to the reaction mixture through a constant-pressure dropping funnel under nitrogen protection. After stirring for 48 h, the mixture was concentrated by rotary evaporation, washed twice with saturated $NaHCO_3$ solution and deionized water successively, dried over anhydrous Na_2SO_4 for 0.5 h, filtered, and distilled under reduced pressure to afford a colorless liquid product (44.1 g, yield 49.3%). The monomer was sealed under nitrogen and stored at -20 °C. The ^{1}H-NMR spectrum is shown in Figure S1. ^{1}H-NMR (400 MHz, Chloroform-*d*): δ 6.46 (d, 1H), 6.15 (dd, 1H), 5.89 (d, 1H), 4.47 (t, 2H), 3.55 (t, 2H).

Synthesis of poly(2-bromoethyl acrylate) (PBrEA)

In a typical polymerization procedure, BrEA monomer (5.1 g, 28.53 mmol) and AIBN (45.5 mg, 0.28 mmol) were dissolved in 2-butanone (30 mL) and transferred to a 50 mL Schlenk flask. The flask was immersed in an oil bath at 70 °C under nitrogen atmosphere and allowed to react for 20 min. The polymerization product was precipitated three times in cold *n*-hexane (-20 °C), filtered, and dried under vacuum at 40 °C for 24 h to afford a colorless viscous solid. The obtained PBrEA samples with different molecular weights were designated as P1–P4. Their number-average molecular weights and dispersities were determined by GPC (with THF as the mobile phase and calibrated with polystyrene standards), and the results are listed in Table S1 and Figure S2. The ^{1}H-NMR spectrum is shown in Figure S3. ^{1}H NMR (400 MHz, Chloroform-*d*) δ 4.40 (s, 2H), 3.82–3.41 (m, 2H), 2.46 (s, 1H), 2.21–1.43 (m, 2H).

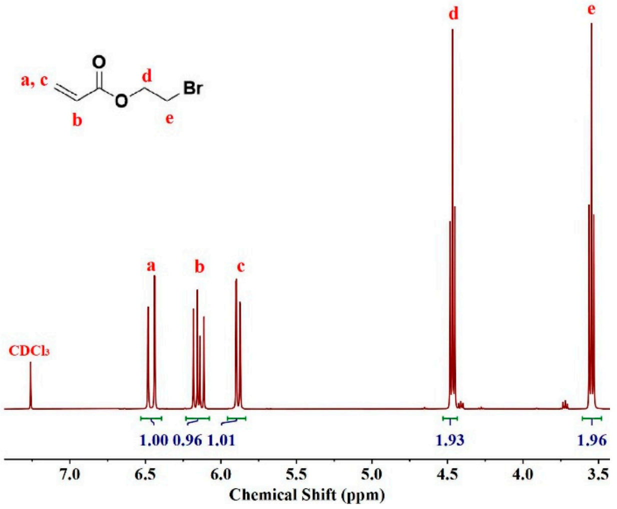

Figure S1 ^{1}H-NMR spectrum of BrEA.

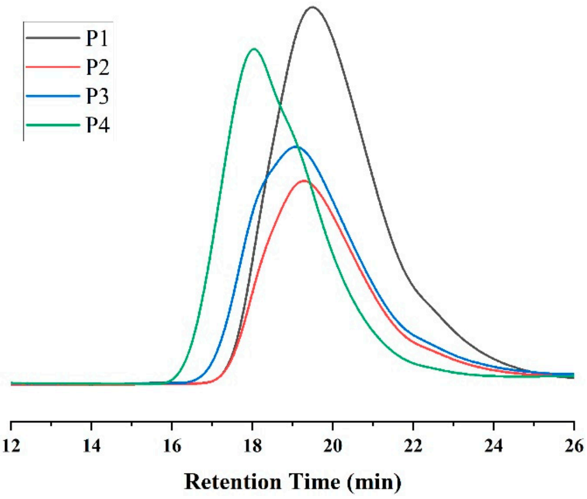

Figure S2 GPC traces of PBrEA samples.

Table S1 Molecular characteristics and polymerization conditions of PBrEA.

Samples	$M_{n,GPC}$ [a] (g/mol)	$Đ$ [b]	BrEA (g, mmol)	AIBN (mg, mmol)	[M]/[I]	V [c] (mL)	Temperature (°C)	Reaction time (min)
P1	34100	1.38	10.0, 55.87	91.7, 0.56	100	60	70	20
P2	47000	1.4	7.8, 43.64	71.1, 0.43	100	40	70	30
P3	53100	1.54	5.0, 27.90	46.1, 0.28	100	25	70	35
P4	68700	1.56	5.1, 28.53	45.5, 0.28	100	30	70	20

[a]Determined by GPC. [b]$Đ = M_w/M_n$, polydispersity index of the sample determined by GPC. [c]Volume of the solvent 2-butanone.

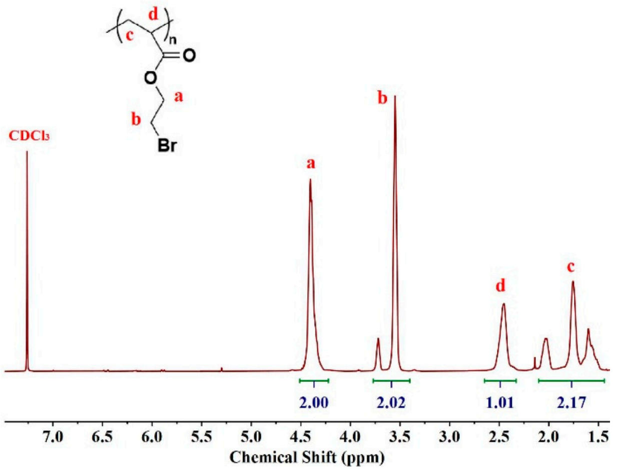

Figure S3 ^{1}H-NMR spectrum of PBrEA sample P4.

Synthesis of 1-alkylimidazoles

Taking 1-octadecylimidazole as an example, 1-bromooctadecane (20.00 g, 60 mmol), imidazole (12.25 g, 180 mmol), and 50 wt% aqueous NaOH solution (18 g, 225 mmol) were dissolved in THF (120 mL) and refluxed at 70 °C for 3 days. After removal of THF by rotary evaporation, the obtained solid was dissolved in chloroform, and the insoluble salts were removed by suction filtration. The filtrate was extracted twice with saturated brine and deionized water successively. The organic phase was dried over anhydrous Na_2SO_4 for 0.5 h, filtered, concentrated by rotary evaporation to remove

chloroform, and dried under vacuum for 24 h to afford a white powder product (16.5 g, yield 85.9%). 1-Dodecylimidazole, 1-eicosylimidazole, and 1-docosylimidazole were synthesized following the same procedure. The ^{1}H-NMR (400 MHz, Chloroform-*d*) data are shown in Figure S4–Figure S7 and Table S2. (Figure S8 shows the expanded methylene region).

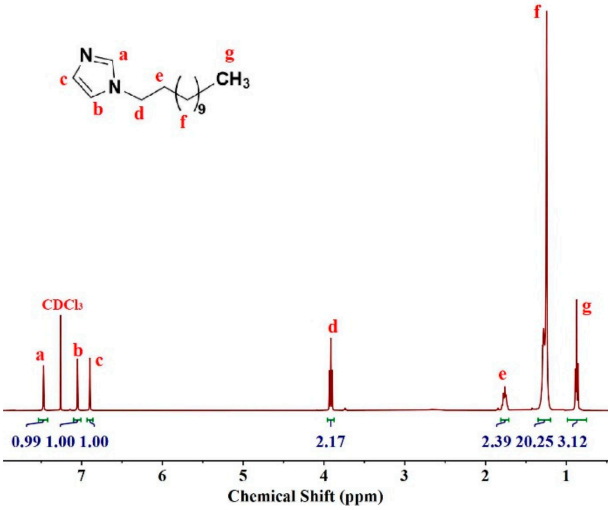

Figure S4 ^{1}H-NMR spectrum of 1-dodecylimidazole.

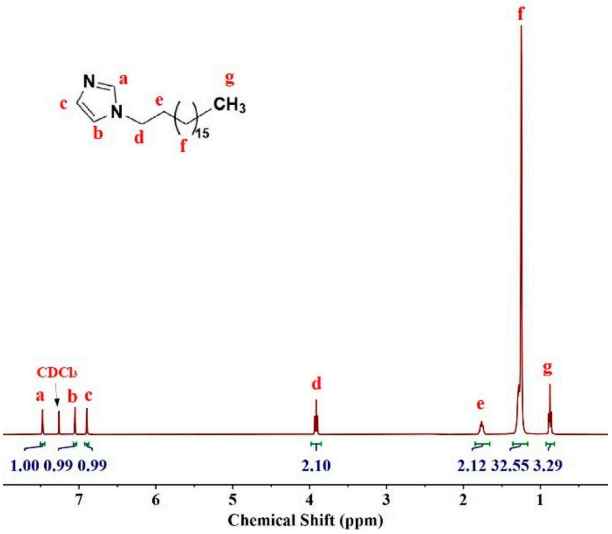

Figure S5 ^{1}H-NMR spectrum of 1-octadecylimidazole.

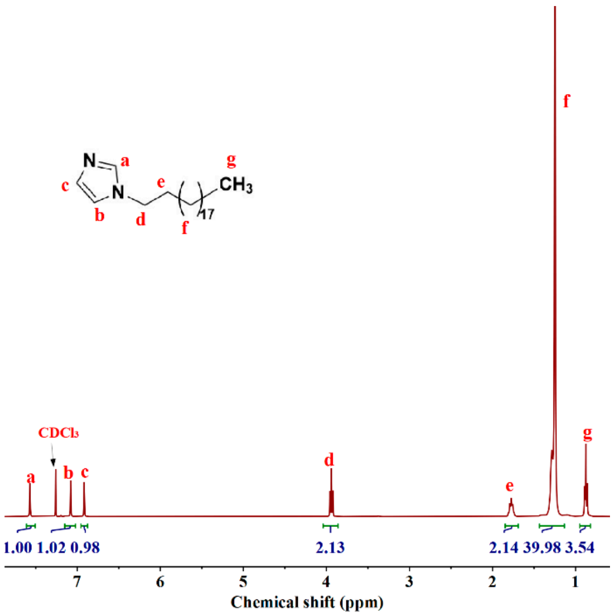

Figure S6 ^{1}H-NMR spectrum of 1-eicosylimidazole.

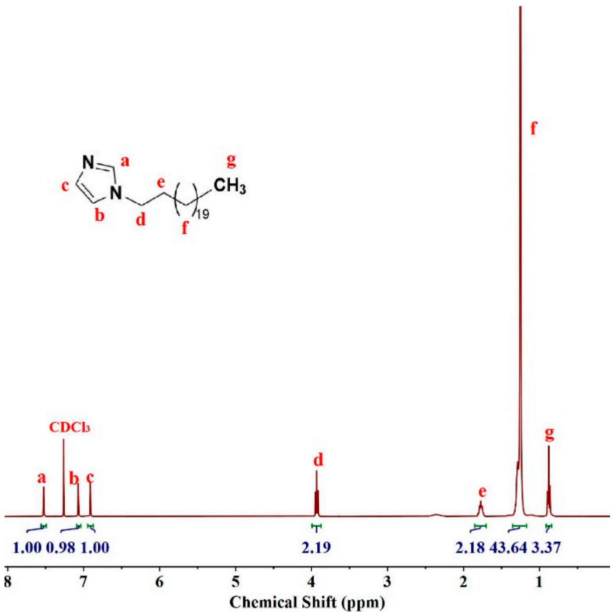

Figure S7 ^{1}H-NMR spectrum of 1-docosylimidazole.

Table S2 Main chemical shifts of individual homologues(δ, ppm).

Compound	Imidazole ring (2,4,5-H)	$-NCH_2-$ (t)	$-CH_2-$ (p)	Methylene envelope (m)	Terminal CH_3- (t)
C12-Im	7.47, 7.05, 6.90	3.92	1.76	1.44 – 1.15 (20H)	0.87
C18-Im	7.50, 7.06, 6.91	3.93	1.77	1.40 – 1.15 (32H)	0.88
C20-Im	7.57, 7.08, 6.92	3.94	1.77	1.43 – 1.11 (40H)	0.88
C22-Im	7.52, 7.07, 6.91	3.93	1.77	1.39 – 1.14 (44H)	0.88

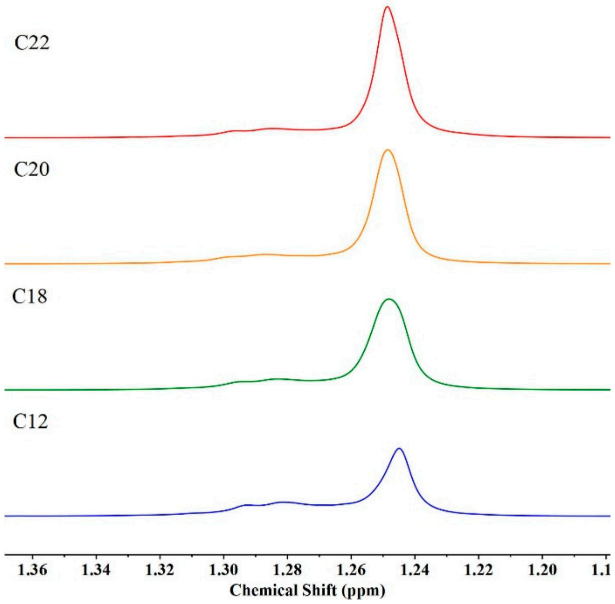

Figure S8 ^{1}H-NMR spectra (expanded methylene region) of the 1-alkylimidazole series (C12–C22).

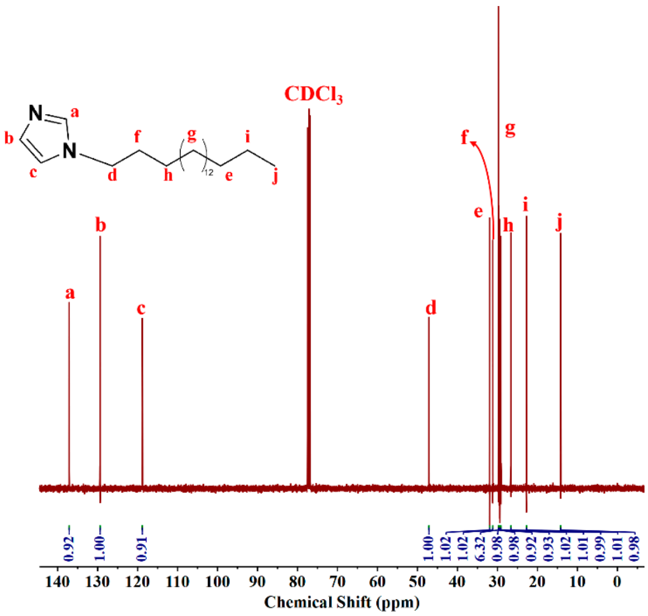

Figure S9 ^{13}C-NMR spectrum of 1-octadecylimidazole.

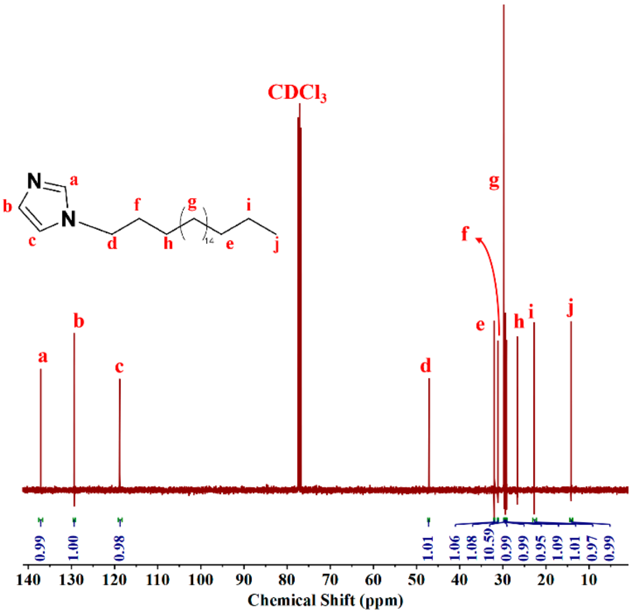

Figure S10 ^{13}C-NMR spectrum of 1-eicosylimidazole.

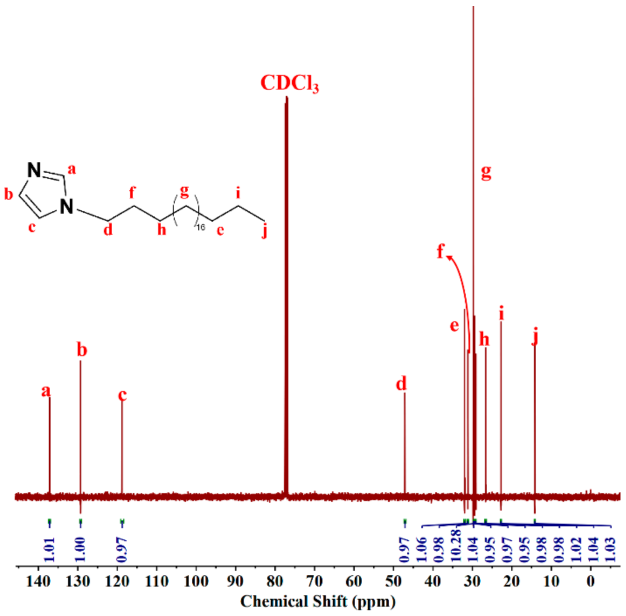

Figure S11 ^{13}C-NMR spectrum of 1-docosylimidazole.

The ^{13}C-NMR spectra of 1-octadecylimidazole, 1-eicosylimidazole, and 1-docosylimidazole were also recorded to confirm the chemical structures(Figures S9 − S11). For these homologues, the most significant difference lies in the number of methylene carbons within the long alkyl chain. The theoretical numbers of methylene carbons in the alkyl chain (excluding the $-NCH_2-$ carbon) are 12, 14, and 16 for the C18, C20, and C22 derivatives, respectively. In the ^{13}C-NMR spectra, the signals corresponding to these methylene carbons appear as a group of overlapping peaks (denoted as peak g). The integrated values obtained by summing these peaks are 12.16, 14.61, and 16.15, which are in good agreement with the theoretical values. The three aromatic carbons of the imidazole ring show consistent chemical shifts and integrations across all three compounds, and the remaining carbon signals (including the $-NCH_2-$, the terminal $-CH_3$, and the α- and β-carbons relative to the imidazole ring) are also well resolved and consistent with the expected structures. These results confirm the successful synthesis of the 1-alkylimidazole intermediates.

Synthesis of $C18_xC8_yB_z$

Taking $C18_{0.60}C8_{0.12}B_{0.28}$ as an example, PBrEA (P4, 0.2 g, 1.12 mmol), 1-octadecylimidazole (1.613 g, 5.04 mmol), and 1-octylimidazole (0.101 g, 0.56 mmol) were dissolved in DMF (10 mL) and transferred to a 25 mL Schlenk flask. The total molar feed ratio of alkylimidazoles to BrEA repeating units was 5:1, and the molar feed ratio of 1-octadecylimidazole to 1-octylimidazole was 9:1. The flask was immersed in an oil bath at 88 °C and stirred under nitrogen atmosphere for 72 h. After the reaction, the mixture was dialyzed against DMF for two days, followed by dialysis against

ethanol for another two days, and then concentrated by rotary evaporation to afford a yellow viscous solid product. The ^{1}H-NMR spectrum is shown in Figure S9. ^{1}H NMR (400 MHz, DMSO-d_6) δ 9.50 (s, 1H), 8.10–7.56 (m, 2H), 4.63–4.15 (m, 4H), 4.00 (s, 2H), 2.37–1.40 (m, 5H), 1.32–1.14 (m, 27H), 0.94–0.72 (m, 3H).

By varying the feed ratio of 1-octadecylimidazole to 1-octylimidazole (r = 0.9–0.3), a series of $C18_xC8_yB_z$ samples with different compositions were obtained.

Characterization of C18$_x$C8$_y$B$_z$

To determine the molar fractions (x, y, z) of each structural unit in the C18$_x$C8$_y$B$_z$ copolymers (with $x + y + z = 1$), a two-step quantitative method was employed based on the integration of the ^{1}H-NMR spectrum (Figure S9).

(1) Determination of the relative proportion of long- and short-chain units (X : Y) in the quaternized segments.

The characteristic resonance at δ = 1.34–1.13 ppm (assigned to peaks g + g′) corresponds to the overlapping signals of the internal methylene protons from the C18 and C8 side chains. In the quaternized repeating units, the C18 chain contributes 30 internal methylene protons (15 × 2 H), while the C8 chain contributes 10 internal methylene protons (5 × 2 H). Let X and Y be the mole fractions of C18 and C8 units among the quaternized segments, respectively (i.e., $X + Y = 1$). Using the integrated value of $I_{g+g'} = 26.70$, the following equation was established:

$$30X + 10(1 - X) = 26.7$$

Solving this equation gives $X = 0.835$ and $Y = 0.165$, indicating that among the quaternized units, the C18-containing units account for 83.5 mol% and the C8-containing units for 16.5 mol%.

(2) Calculation of the overall mole fractions (x, y, z).

The degree of quaternization (η) was determined from the ratio of the imidazolium proton peaks (i + j, integral 2.02) to the total imidazole ring proton peaks (a + b, integral 5.52):

$$\eta = 2I_{i+j} / I_{a+b} = 2 \times 2.02 / 5.52 \approx 0.725$$

Thus, the final mole fractions of each component in the copolymer are:

$$x = \eta \times X = 0.725 \times 0.835 \approx 0.605$$

$$y = \eta \times Y = 0.725 \times 0.165 \approx 0.120$$

$$z = 1 - \eta = 1 - 0.725 = 0.275$$

The calculated composition is therefore C18$_{0.60}$C8$_{0.12}$B$_{0.28}$. The actual compositions of other copolymers in this series were calculated in the same manner from their ^{1}H-NMR integration data. The detailed information of the C18$_x$C8$_y$B$_z$ samples is listed in Table S3, and the ^{1}H-NMR spectra of this series are shown in Figure S10−Figure S16.

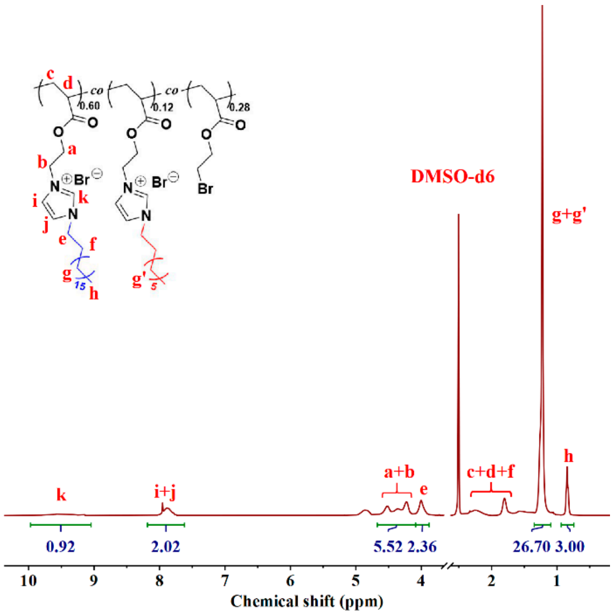

Figure S12 ^{1}H-NMR spectrum of $C18_{0.60}C8_{0.12}B_{0.28}$.

Table S3 Molecular characteristics of $C18_xC8_yB_z$.

Samples	Precursor	r	Degree of quaternization[a] η (%)	x	y	z
$C18_{0.78}C8_{0.15}B_{0.07}$	P1	0.9	93.02	0.78	0.15	0.07
$C18_{0.60}C8_{0.12}B_{0.28}$		0.9	72.46	0.60	0.12	0.28
$C18_{0.54}C8_{0.19}B_{0.27}$		0.8	72.59	0.54	0.19	0.27
$C18_{0.47}C8_{0.27}B_{0.26}$		0.7	74.21	0.47	0.27	0.26
$C18_{0.38}C8_{0.38}B_{0.24}$	P4	0.6	75.90	0.38	0.38	0.24
$C18_{0.30}C8_{0.43}B_{0.27}$		0.5	73.26	0.30	0.43	0.27
$C18_{0.23}C8_{0.49}B_{0.28}$		0.4	72.46	0.23	0.49	0.28
$C18_{0.20}C8_{0.53}B_{0.27}$		0.3	73.39	0.20	0.53	0.27

[a]Degrees of quaternization of BrEA units were calculated from the ^{1}H-NMR spectra. [b]Molar fraction of the 1-octadecylimidazole (C18) side chain relative to the total alkylimidazole side chains, calculated from the ^{1}H-NMR spectra.

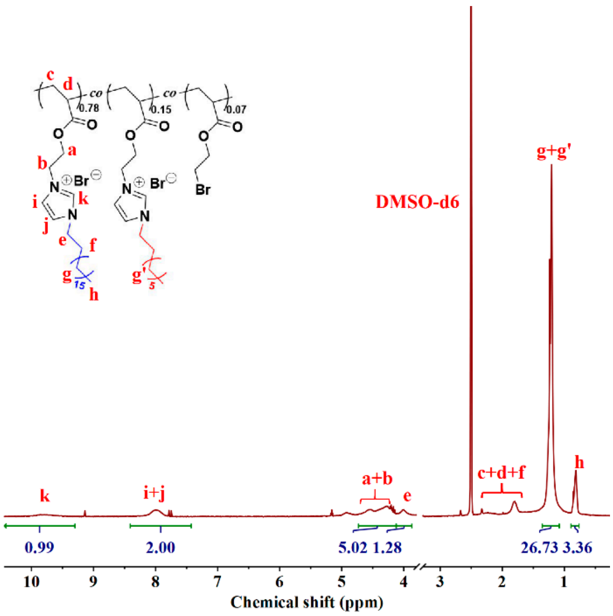

Figure S13 ^{1}H-NMR spectrum of $C18_{0.78}C8_{0.15}B_{0.07}$.

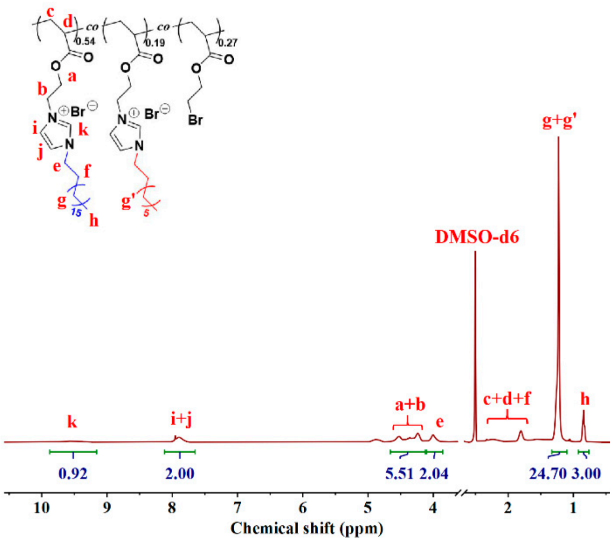

Figure S14 ^{1}H-NMR spectrum of $C18_{0.54}C8_{0.19}B_{0.27}$.

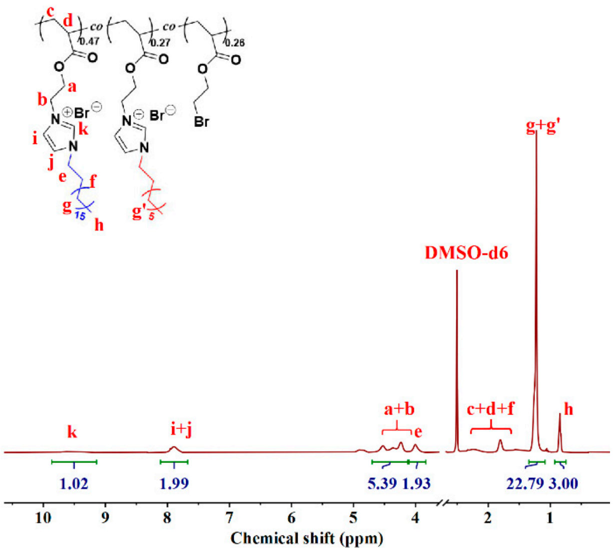

Figure S15 ^{1}H-NMR spectrum of $C18_{0.47}C8_{0.27}B_{0.26}$.

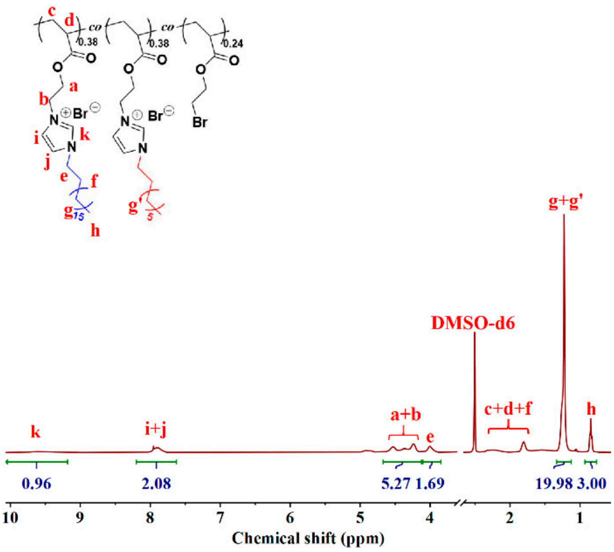

Figure S16 ^{1}H-NMR spectrum of $C18_{0.38}C8_{0.38}B_{0.24}$.

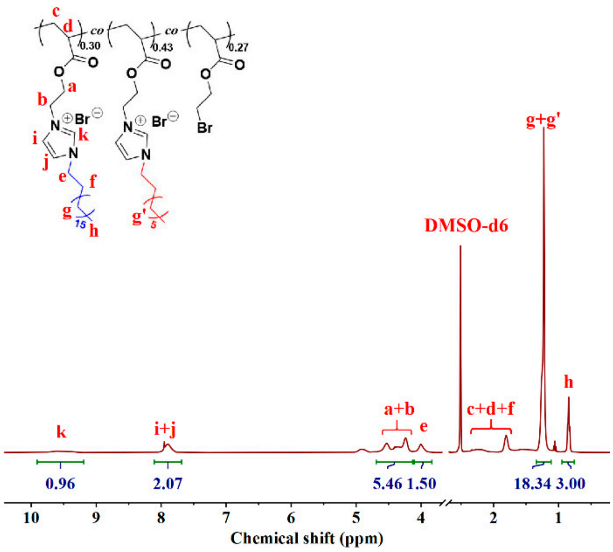

Figure S17 ^{1}H-NMR spectrum of $C18_{0.30}C8_{0.43}B_{0.27}$.

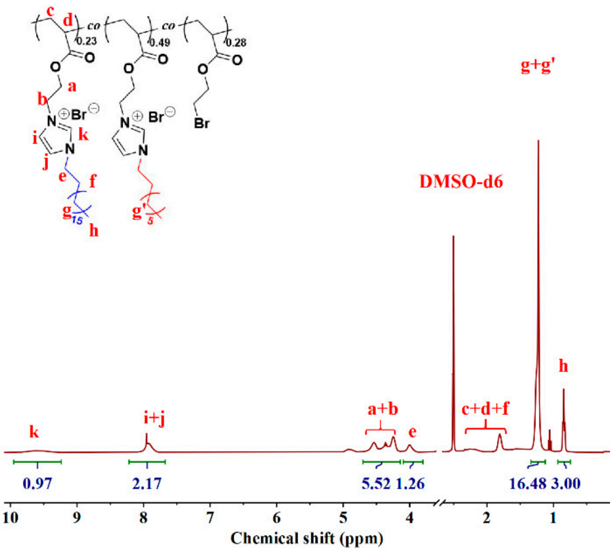

Figure S18 ^{1}H-NMR spectrum of $C18_{0.23}C8_{0.49}B_{0.28}$.

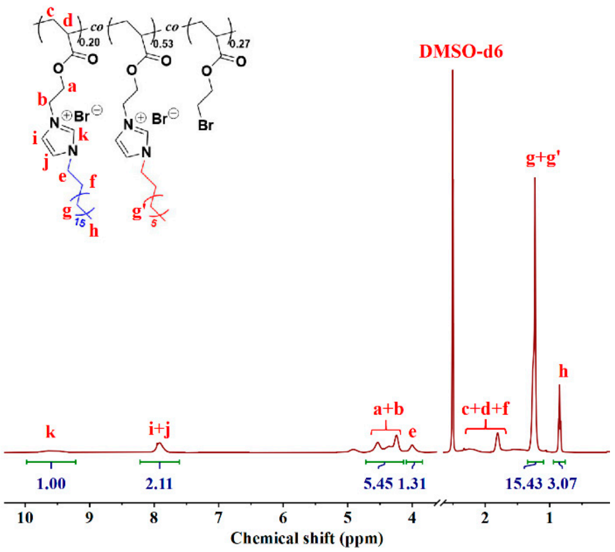

Figure S19 ^{1}H-NMR spectrum of $C18_{0.20}C8_{0.53}B_{0.27}$.

Synthesis of $C20_rC12_{1\text{-}r}$ and $C22_rC12_{1\text{-}r}$

The $C20_rC12_{1\text{-}r}$ series was prepared by quaternization of P2 with 1-eicosylimidazole and 1-dodecylimidazole, while the $C22_rC12_{1\text{-}r}$ series was obtained from P3 with 1-docosylimidazole and 1-dodecylimidazole. The feed ratio (r), reaction conditions, and workup procedures were identical to those for the $C18_xC8_yB_z$ series.

However, as the side chain length of the quaternized units increased, the solubility of the $C20_rC12_{1\text{-}r}$ and $C22_rC12_{1\text{-}r}$ copolymers in DMSO decreased significantly (Figure S17), resulting in the absence of well-resolved characteristic proton signals in their ^{1}H-NMR spectra (Figure S18); only a single broad multiplet at δ = 4.24–4.05 ppm was observed, which was tentatively assigned to the methylene protons ($-OCH_2CH_2N-$) connecting the ester group and the imidazolium ring. Accordingly, in the subsequent study, the samples of these two series are denoted by the feed ratio of the long-chain alkylimidazole, i.e., $C20_rC12_{1\text{-}r}$ and $C22_rC12_{1\text{-}r}$.

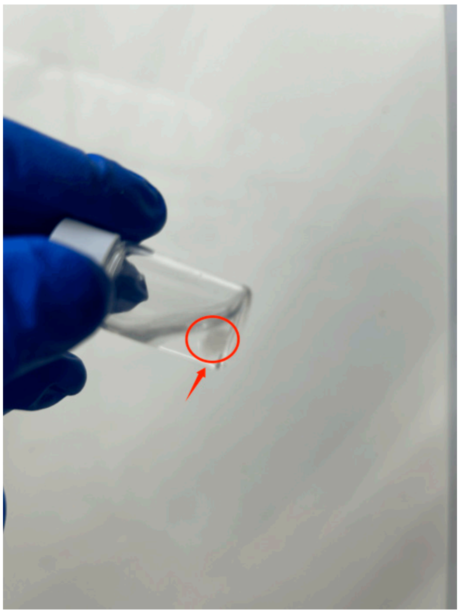

Figure S20 Solubility of $C22_{0.5}C12_{0.5}$ in DMSO solvent.

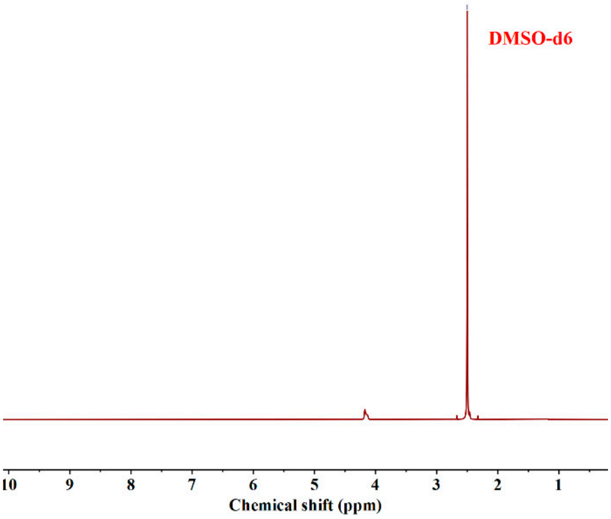

Figure S21 ^{1}H-NMR spectrum of $C22_{0.5}C12_{0.5}$.

Part 2: Phase behaviors of the comb-like copolymers

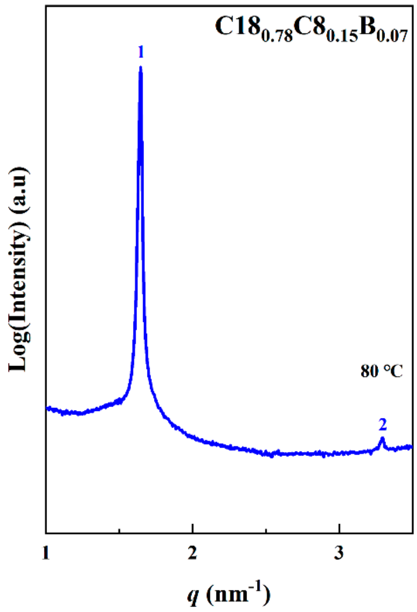

Figure S22 SAXS profiles of $C18_{0.78}C8_{0.15}B_{0.07}$ at 80 °C.

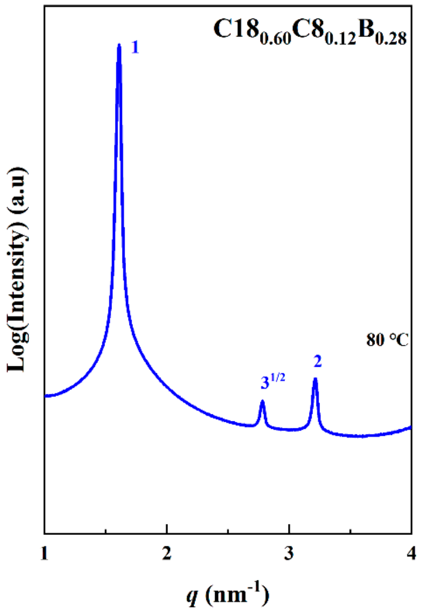

Figure S23 SAXS profiles of $C18_{0.60}C8_{0.12}B_{0.28}$ at 80 °C.

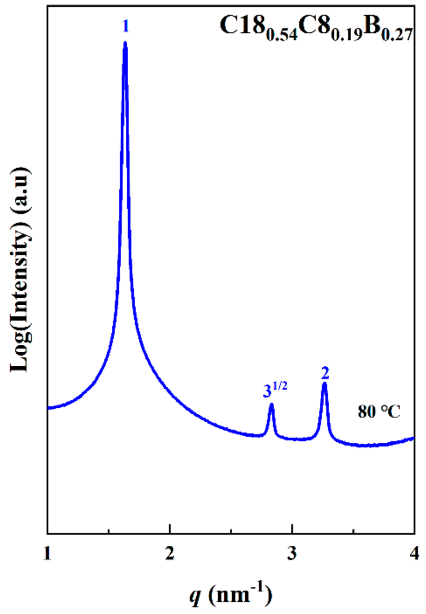

Figure S24 SAXS profiles of $C18_{0.54}C8_{0.19}B_{0.27}$ at 80 °C.

Table S4 Representative Lorentz peak fitting parameters for the primary SAXS peak of $C18_{0.78}C8_{0.15}B_{0.07}$ at selected temperatures.

Temperature (°C)	y_0 (a.u.)	q^*, x_c (nm^{-1})	FWHM, w (nm^{-1})	Error of w (nm^{-1})	A (a.u)	Intensity, H (a.u)	Error of H (a.u)
30	2.171	1.546	0.390	0.016	0.405	0.662	0.013
40	3.201	1.568	0.272	0.010	0.331	0.776	0.015
50	3.945	1.613	0.177	0.009	0.267	0.960	0.026
60	5.000	1.645	0.055	0.002	0.129	1.508	0.039
70	5.998	1.644	0.048	0.002	0.121	1.618	0.036
80	6.998	1.644	0.048	0.002	0.121	1.618	0.036
90	8.006	1.623	0.043	0.001	0.116	1.697	0.038
100	9.013	1.611	0.043	0.002	0.115	1.709	0.040
110	10.019	1.607	0.041	0.002	0.111	1.723	0.043
120	11.022	1.605	0.041	0.001	0.110	1.731	0.042
130	12.027	1.604	0.039	0.001	0.108	1.745	0.043
140	13.035	1.601	0.038	0.001	0.105	1.768	0.045
150	14.036	1.602	0.038	0.001	0.105	1.773	0.044
160	15.038	1.604	0.037	0.001	0.104	1.781	0.043
170	16.040	1.606	0.036	0.001	0.102	1.790	0.042
180	17.040	1.608	0.035	0.001	0.100	1.805	0.042

Peak fitting of the SAXS primary scattering profiles was performed using the built-in Lorentz function in Origin software (System Fit). The fitting equation is: $y = y_0 + (2A/\ \pi)$

($w/(4(x-x_c)^2 + w^2)$)). In the Lorentz fitting function, the parameters w and H correspond to the full width at half-maximum (FWHM) and the peak height (Intensity), respectively. The intensity values used for analysis are peak heights (H), not integrated areas (A). All fits converged successfully. Due to the 10 °C temperature step in the SAXS measurements, the reported values are representative rather than exhaustive for all temperatures.

Table S5 Assignments of the scattering peaks for the $C18_{0.47}C8_{0.27}B_{0.26}$.

Miller Indices (hkl)	q_{obs} (nm^{-1})	q_{calc} (nm^{-1})	%Residual ($\Delta q/q_{calc}\times 100$)
(110)	0.950	0.950	0.00
(200)	1.354	1.344	0.76
(210)	1.509	1.502	0.44
(211)	1.644	1.646	-0.14
(220)	1.905	1.900	0.22
(310)	2.125	2.125	0.00
(222)	2.329	2.328	0.05
(320)	2.431	2.423	0.33
(321)	2.516	2.514	0.09
(400)	2.692	2.688	0.15
(420)	3.010	3.005	0.16
(421)	3.079	3.079	0.00
(422)	3.291	3.292	-0.02

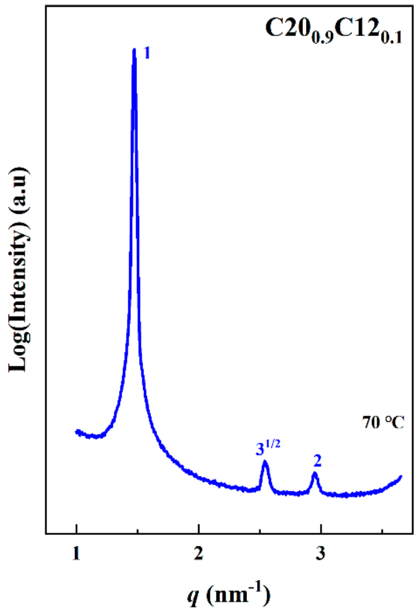

Figure S25 SAXS profiles of C20$_{0.9}$C12$_{0.1}$ at 70 °C.

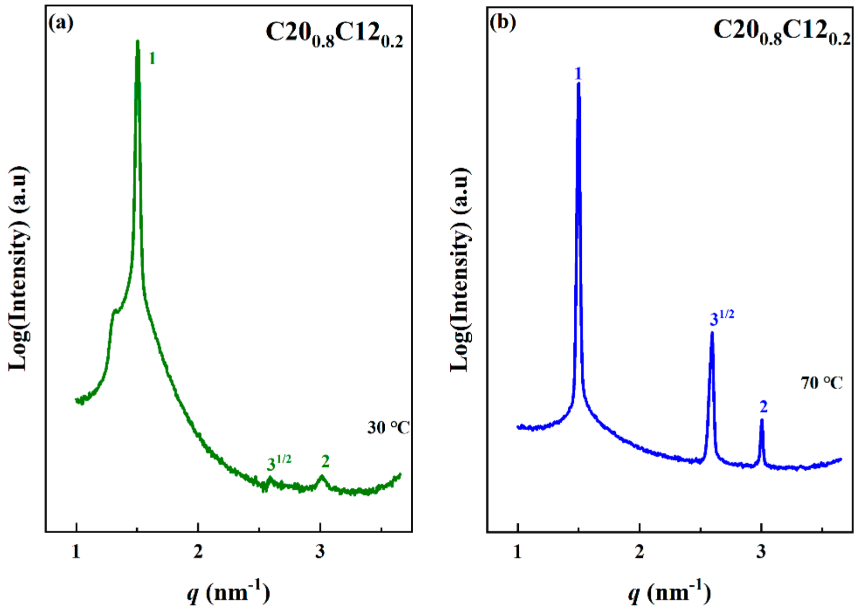

Figure S26 SAXS profiles of C20$_{0.8}$C12$_{0.2}$ at (a) 30 °C. and (b) 70 °C.

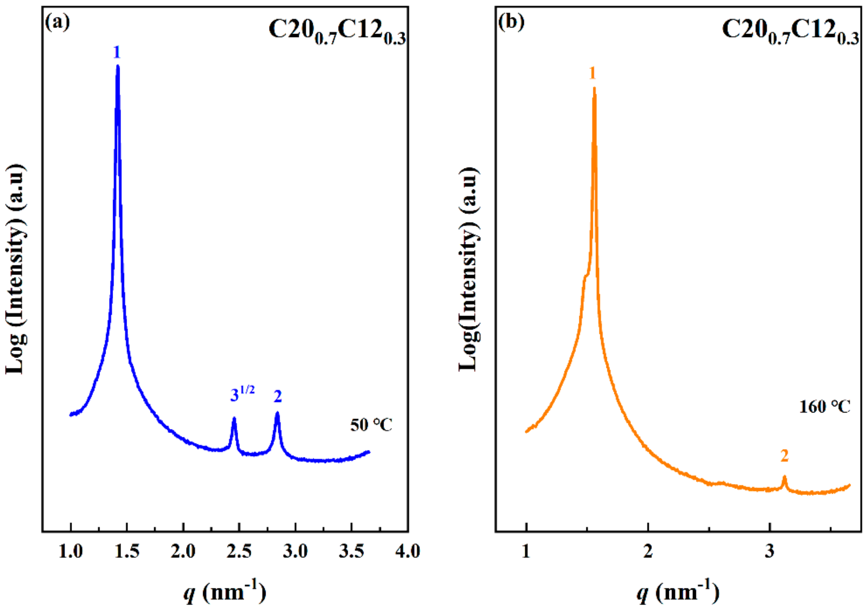

Figure S27 SAXS profiles of C20$_{0.7}$C12$_{0.3}$ at (a) 50 °C and (b) 160 °C.

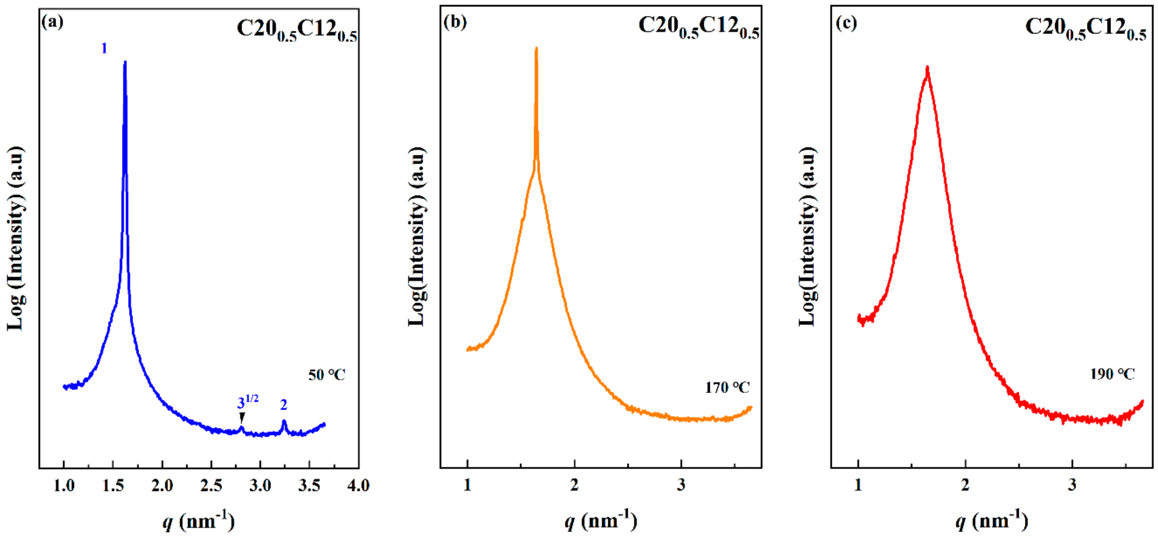

Figure S28 SAXS profiles of C20$_{0.5}$C12$_{0.5}$ at (a) 50 °C, (b) 170 °C and (c) 190 °C.

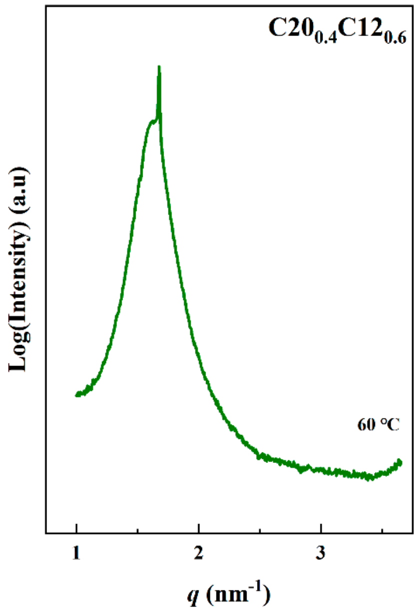

Figure S29 SAXS profiles of C20$_{0.4}$C12$_{0.6}$ at 60 °C.

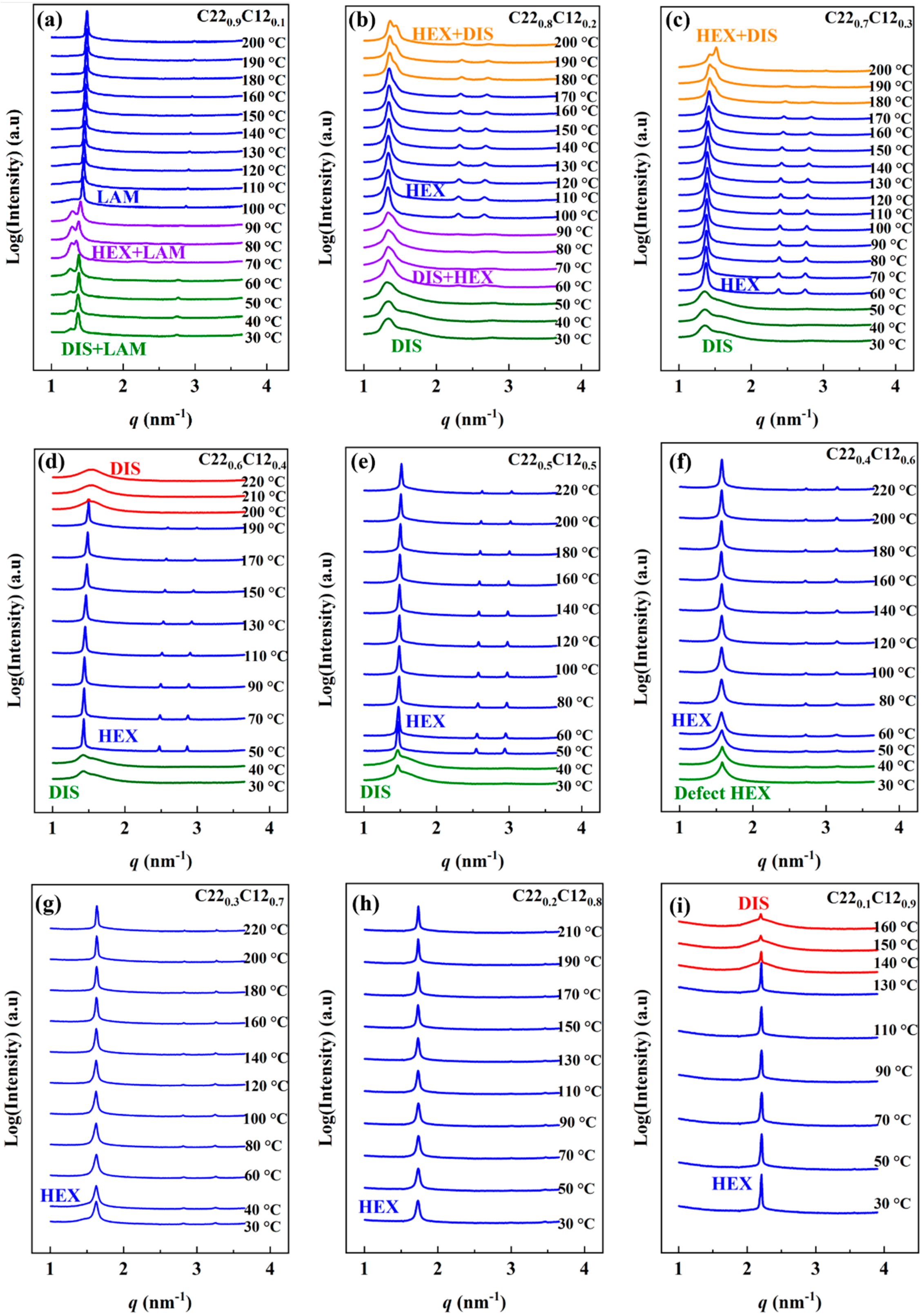

Figure S30 Temperature-variable SAXS profiles of (a) C22$_{0.9}$C12$_{0.1}$, (b) C22$_{0.8}$C12$_{0.2}$, (c) C22$_{0.7}$C12$_{0.3}$, (d) C22$_{0.6}$C12$_{0.4}$, (e) C22$_{0.5}$C12$_{0.5}$, (f) C22$_{0.4}$C12$_{0.6}$, (g) C22$_{0.3}$C12$_{0.7}$, (h) C22$_{0.2}$C12$_{0.8}$, and (i) C22$_{0.1}$C12$_{0.9}$.

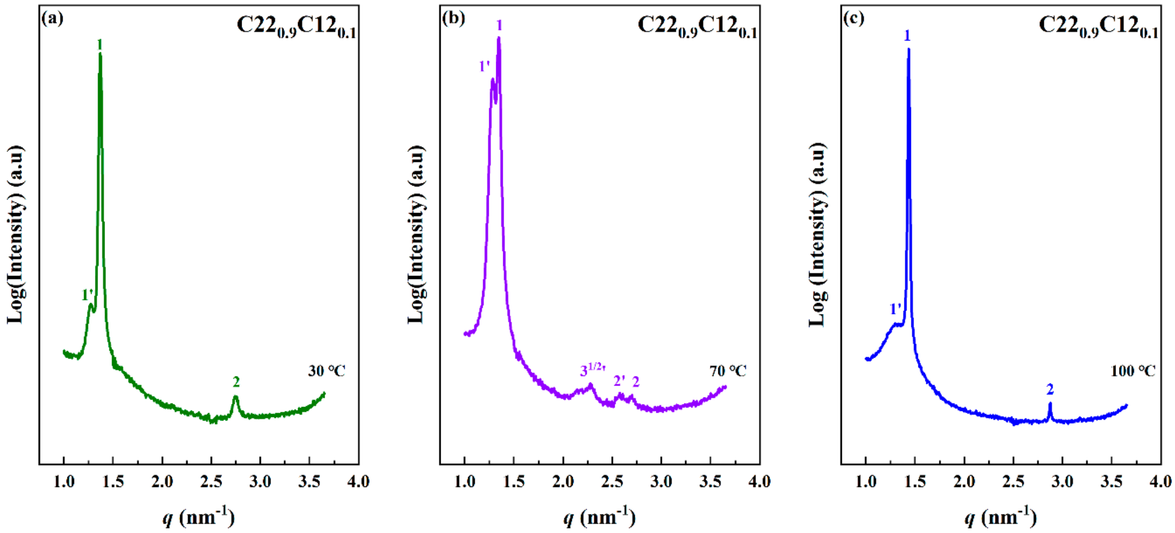

Figure S31 SAXS profiles of C22$_{0.9}$C12$_{0.1}$ at (a) 30 °C, (b) 70 °C and (c) 100 °C.

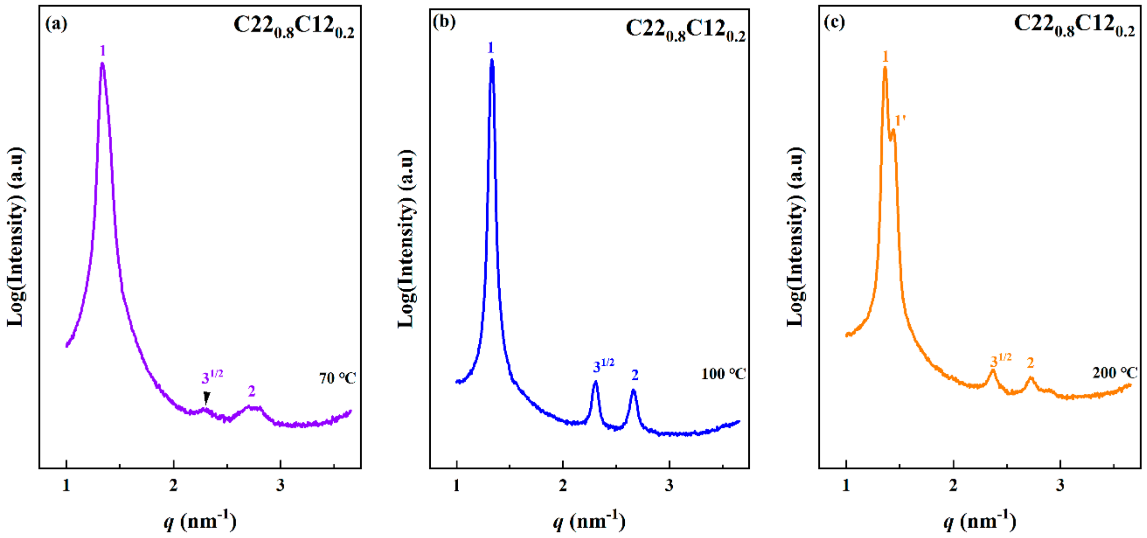

Figure S32 SAXS profiles of C22$_{0.8}$C12$_{0.2}$ at (a) 70 °C, (b) 100 °C and (c) 200 °C.

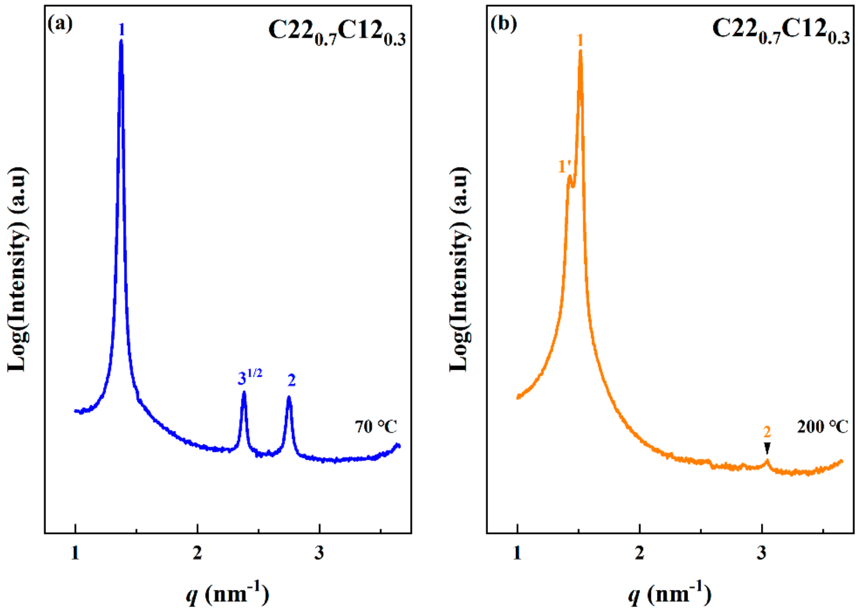

Figure S33 SAXS profiles of C22$_{0.7}$C12$_{0.3}$ at (a) 70 °C and (b) 200 °C.

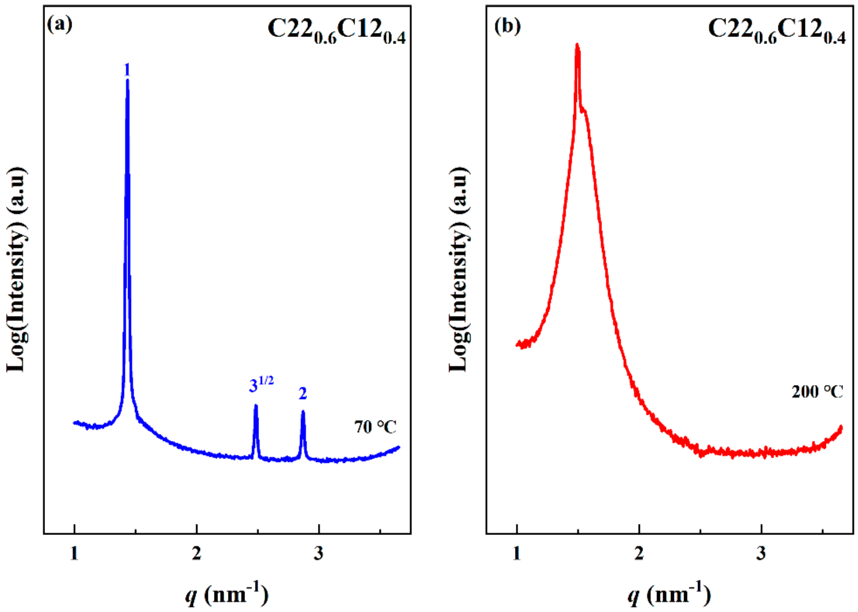

Figure S34 SAXS profiles of C22$_{0.6}$C12$_{0.4}$ at (a) 70 °C and (b) 200 °C.

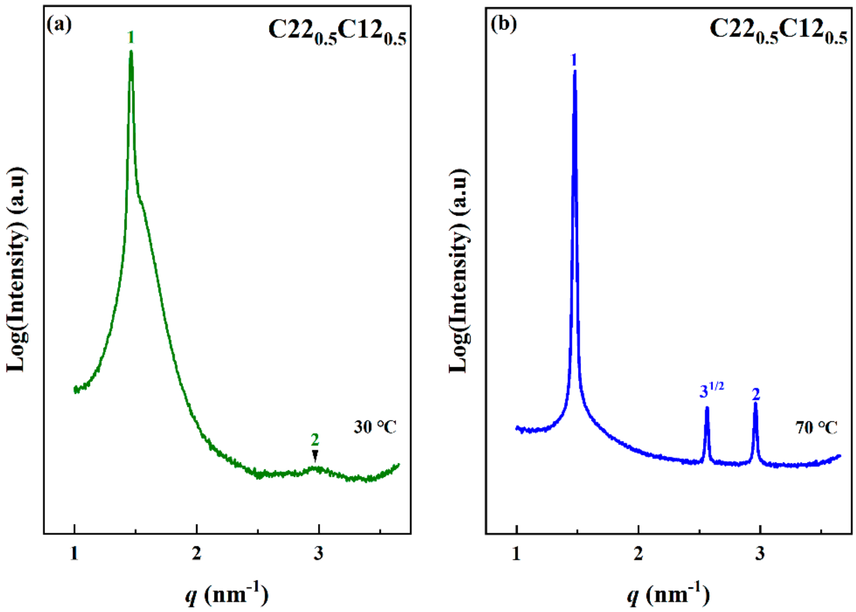

Figure S35 SAXS profiles of $C22_{0.5}C12_{0.5}$ at (a) 30 °C and (b) 70 °C.

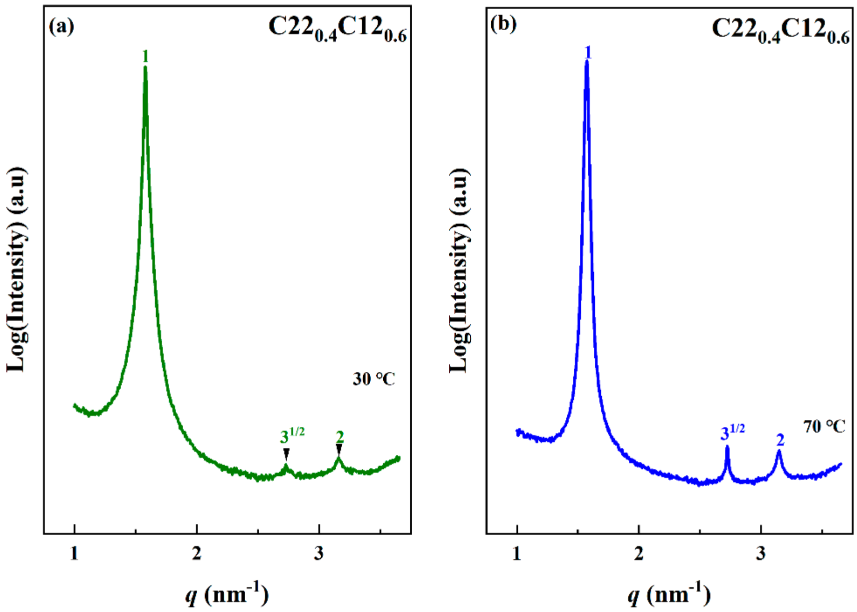

Figure S36 SAXS profiles of $C22_{0.4}C12_{0.6}$ at (a) 30 °C and (b) 70 °C.

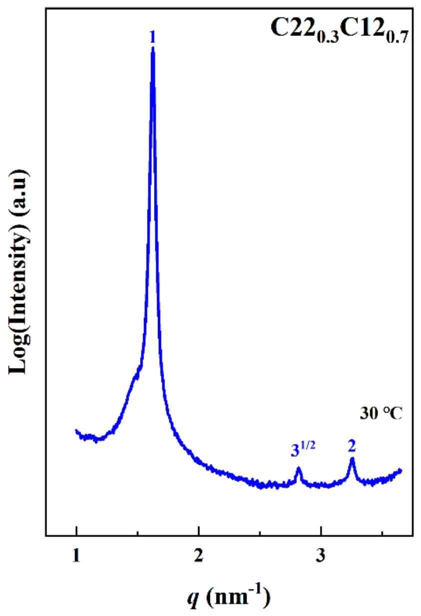

Figure S37 SAXS profile of C22$_{0.3}$C12$_{0.7}$ at 30 °C.

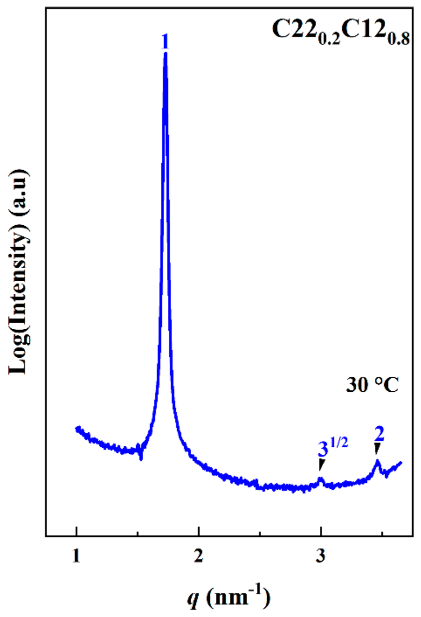

Figure S38 SAXS profile of C22$_{0.2}$C12$_{0.8}$ at 30 °C.

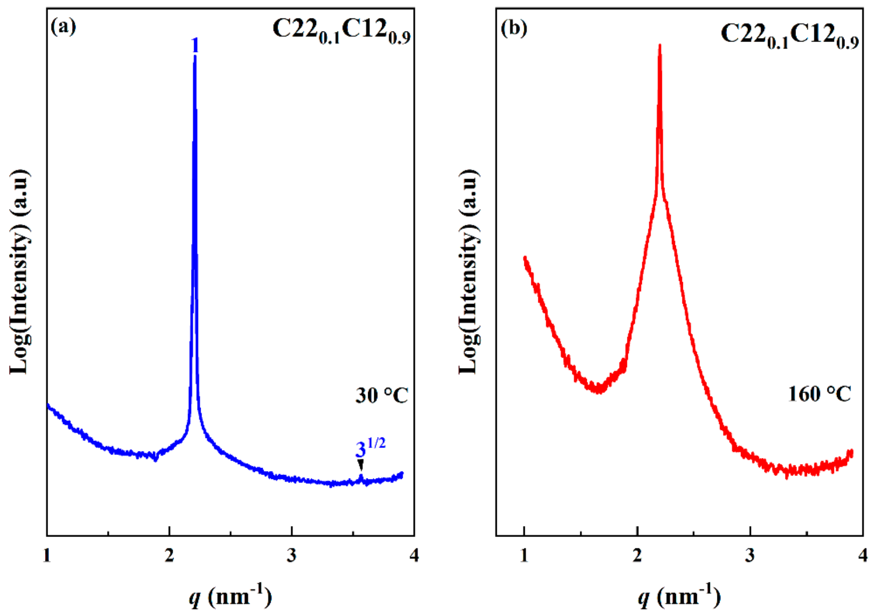

Figure S39 SAXS profiles of C22$_{0.1}$C12$_{0.9}$ at (a) 30 °C and (b) 160 °C.

The FWHM ~ temperature plots (Figure S37) further corroborate the phase transition assignments: for closed-loop samples (e.g., $r = 0.8 - 0.6$), the FWHM first drops sharply at T_{DOT} and then increases again at higher temperatures corresponding to UODT. Notably, as r decreases, T_{DOT} exhibits a clear decreasing trend, consistent with the lowered T_{m} (Figure S38). It should be noted that the FWHM data for C22$_{0.9}$C12$_{0.1}$ are not included in Figure S37, because the coexistence of ordered and disordered phases leads to significant splitting of the primary peak, making the fitting results unreliable. For $r = 0.1$, the FWHM shows an abrupt increase at elevated temperatures, consistent with UODT. These results are fully consistent with the SAXS observations and further support the assignment of phase behaviors for the C22$_r$C12$_{1-r}$ series.

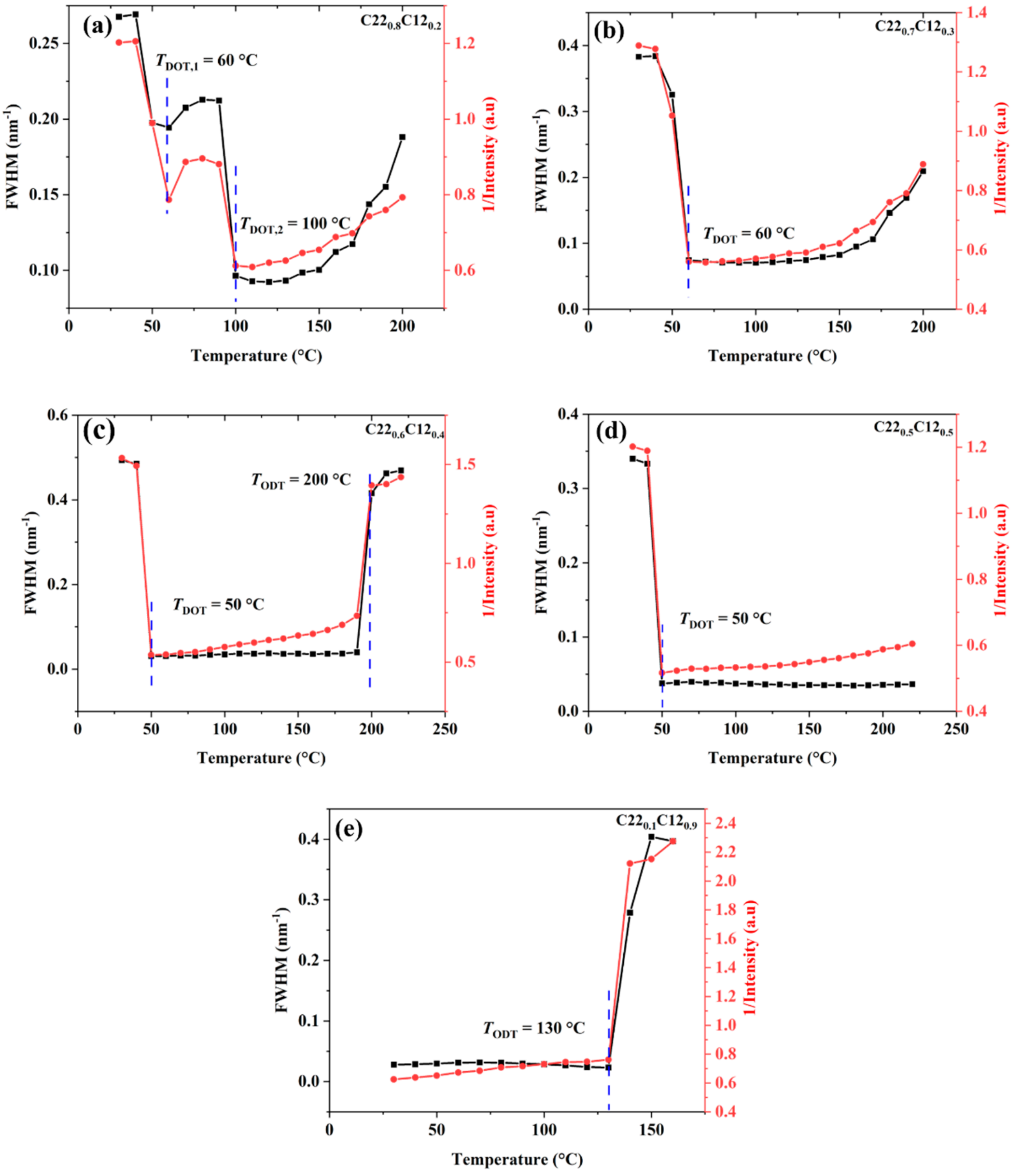

Figure S40 FWHM (black squares) and 1/Intensity (red circles) of the SAXS primary peaks at various temperatures upon heating for (a) C22$_{0.8}$C12$_{0.2}$, (b) C22$_{0.7}$C12$_{0.3}$, (c) C22$_{0.6}$C12$_{0.4}$, (d) C22$_{0.5}$C12$_{0.5}$, and (e) C22$_{0.1}$C12$_{0.9}$.

Part 3: DSC and TGA curves of comb-like copolymers

To further confirm the phase transition behavior observed by SAXS, DSC measurements were performed on the C22$_r$C12$_{1-r}$ samples, and the FWHM of the SAXS primary peak was analyzed as a function of temperature (Figures S28 and S38). The melting temperatures (T_m) and final melting temperatures (T_{fm}) extracted from the DSC curves are summarized in Table 1, along with the T_{DOT} values determined by SAXS. For samples exhibiting LDOT (r = 0.9 – 0.5), the T_{fm} values correlate well with the T_{DOT} determined from SAXS (Table 1), confirming that the LDOT originates from the melting of side-chain crystallites. For example, for the C22$_{0.9}$C12$_{0.1}$ sample, the DSC curve exhibits a melting peak with T_m = 58.5 °C and T_{fm} = 72.3 °C, while the T_{DOT} determined from SAXS is 70 °C.

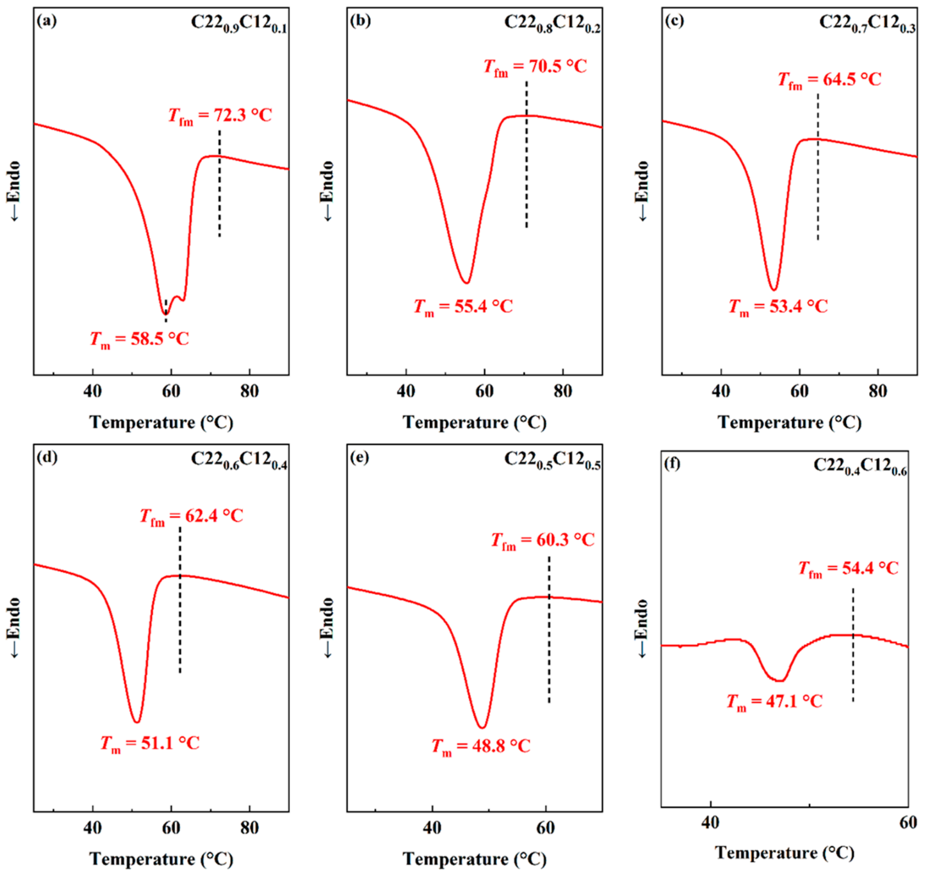

Figure S41 The DSC curves of (a) C22$_{0.9}$C12$_{0.1}$, (b) C22$_{0.8}$C12$_{0.2}$, (c) C22$_{0.7}$C12$_{0.3}$, (d) C22$_{0.6}$C12$_{0.4}$ (e) C22$_{0.5}$C12$_{0.5}$ and (f) C22$_{0.4}$C12$_{0.6}$.

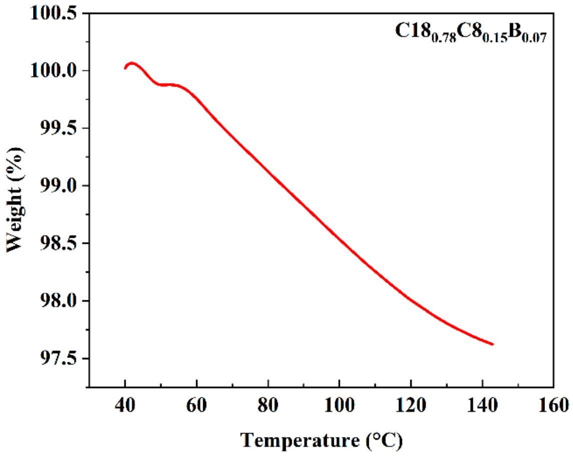

Figure S42 TGA curve of C18$_{0.78}$C8$_{0.15}$B$_{0.07}$ from room temperature to 140 °C under N_2 atmosphere.

A slight initial mass increase was observed in the TGA curve (Figure S39), which is a well-known instrumental artifact primarily caused by the buoyancy effect.
